# Theoretical Analysis of a Mathematical Relation between Driving Pressures in Membrane-Based Desalting Processes

**DOI:** 10.3390/membranes11030220

**Published:** 2021-03-19

**Authors:** Sung Ho Chae, Joon Ha Kim

**Affiliations:** 1 School of Earth Sciences and Environmental Engineering, Gwangju Institute of Science and Technology (GIST), Gwangju 61005, Korea; kha5s@gm.gist.ac.kr; 2 International Environmental Research Institute, Gwangju Institute of Science and Technology (GIST), Gwangju 61005, Korea

**Keywords:** forward osmosis, pressure-retarded osmosis, reverse osmosis, osmotic pressure, hydraulic pressure

## Abstract

Osmotic and hydraulic pressures are both indispensable for operating membrane-based desalting processes, such as forward osmosis (FO), pressure-retarded osmosis (PRO), and reverse osmosis (RO). However, a clear relation between these driving pressures has not thus far been identified; hence, the effect of change in driving pressures on systems has not yet been sufficiently analyzed. In this context, this study formulates an actual mathematical relation between the driving pressures of membrane-based desalting processes by taking into consideration the presence of energy loss in each driving pressure. To do so, this study defines the pseudo-driving pressures representing the water transport direction of a system and the similarity coefficients that quantify the energy conservation rule. Consequently, this study finds three other theoretical constraints that are required to operate membrane-based desalting processes. Furthermore, along with the features of the similarity coefficients, this study diagnoses the commercial advantage of RO over FO/PRO and suggests desirable optimization sequences applicable to each process. Since this study provides researchers with guidelines regarding optimization sequences between membrane parameters and operational parameters for membrane-based desalting processes, it is expected that detailed optimization strategies for the processes could be established.

## 1. Introduction

Desalination processes, such as forward osmosis (FO) and reverse osmosis (RO), have contributed to relieving global water stress [1,2,3]. Moreover, renewable energy-generating processes that use basic mechanisms of desalination, such as pressure-retarded osmosis (PRO), are being vigorously researched to help cope with energy shortages in the era of climate change [4,5]. In spite of their different objectives, FO, RO, and PRO are collectively called membrane-based desalting processes (or systems) because they all commonly utilize a “desalting mechanism” with semi-permeable membranes.

A fundamental mechanism of membrane-based desalting processes is to harness a balance between osmotic pressure and hydraulic pressure. Since osmotic pressure and hydraulic pressure are key components for running membrane-based desalting processes, they are called driving pressures. In membrane-based desalting processes, these driving pressures are exerted in opposite directions. Osmotic pressure allows water molecules to shift from a less concentrated side to a more concentrated side [6]. Hydraulic pressure, on the other hand, forces water molecules to shift from the more concentrated side to the less concentrated side. In the ideal case, water transport should cease if the magnitudes of osmotic pressure and hydraulic pressure become identical [7,8]. However, it is widely understood that such a situation does not occur in practical systems—namely, 1 bar of osmotic pressure and 1 bar of hydraulic pressure do not have the same impact on determining the performance of membrane-based desalting systems even though their magnitude on a pressure gauge is identical. Despite the popular perception that these driving pressures do not have the same effects on different types of membrane-based desalting systems, theoretical studies on this topic have seldom been conducted. Most recent studies have focused on the impacts of driving pressures on membrane parameters [9,10,11,12,13] or on the dependence of osmotic pressure on system temperature [14,15]. Since the mathematical relation between driving pressures can clearly help optimize membrane-based desalting systems and allow for an analysis of the impacts of driving pressures on membrane-based desalting systems, finding this relation is highly advisable.

However, the relation is difficult to determine directly because there are a variety of equations for the osmotic pressure, showing drastic discrepancies. Although the van’t Hoff equation, which was formulated in the 19th century, shows high goodness of fit in a region of low solute concentration, there is a significant gap between the real osmotic pressure values and the results from the van’t Hoff equation in a high solute concentration region. To prevent such a gap between the real values and the calculated values, a coefficient appended to a solute concentration term in the van’t Hoff equation should be iteratively changed [16]. Without iterative updates for the coefficient, a gap occurs between the real values and the calculated values. According to a previous study [17], the maximum deviation of the linear osmotic pressure approximation is estimated as 6.8% in the salinity range of 0–70 g/kg. In this context, various equation and model types have been devised and revised in order to accurately measure the value of osmotic pressure. However, every extant model or equation has drawbacks—for example, the solute concentration of the relevant solution must be sufficiently low [18] or the solute must not be electrolytic [19]. A previous study derived a new osmotic pressure equation that does not require extra constraints. Still, the mathematical results from the study also need to be experimentally demonstrated by various types of solutions [20]. After all, accurate values of the osmotic pressure are as yet uncertain. In particular, the value of osmotic pressure in a high solute concentration region significantly differs from model to model [19,21]. In this regard, researchers have recently implemented molecular dynamics or density function theory to obtain a relatively accurate value of the osmotic pressure [22]. These models estimate the value of osmotic pressure by simulating the repulsive and attractive forces among the molecules in a given solution. Although relatively accurate values can be estimated with such methods, the factual equation for osmotic pressure remains elusive because the techniques used to obtain the values are highly dependent on quantum mechanics rather than deterministic models. Hence, a detour measure should be taken to find the relation between driving pressures in membrane-based desalting systems.

In this context, the objective of the current study is to formulate the mathematical relation between driving pressures in membrane-based desalting systems. To formulate the mathematical relation of driving pressures, this study utilizes new coefficients that allow for an explanation of the energy conservation rule. The new coefficients play a role in establishing the difference from the conventional models of membrane-based desalting systems. With the redefined model and new coefficients, this study represents the overall tendencies of membrane-based desalting processes in accordance with the ratio of the driving pressures; it also discusses the implications of the results applicable to actual membrane-based desalting processes.

## 2. Materials and Methods 

### 2.1. Postulates for Membrane-Based Desalting Systems

To establish a newly defined model that can elucidate the relation between osmotic pressure and hydraulic pressure, self-evident postulates are required. There are seven such postulates, each of which is necessary for determining the features of the new model. Note that every postulate that appears in this paper is denoted as postulate (P.*) after being introduced as Postulate # for the sake of brevity and convenience. The first postulate concerns the relation between driving pressures and water transport.

**Postulate** **1.** *The magnitude of water transport across a semi-permeable membrane must increase if the pressure that drives the water transport keeps increasing.* (P.1)

For example, if water molecules are transported across a semi-permeable membrane from a more concentrated side to a less concentrated side with 0.01 m^3^/min of volumetric flux at 10 bar of hydraulic pressure, then the magnitude of volumetric flux at 13 bar, 16 bar, and 20 bar must be higher than 0.01 m^3^/min. As the driving pressure increases, the magnitude of water transport never decreases—or, at least, remains unchanged—as long as the direction of water transport is not reversed. That is, postulate (P.1) implies that the plot of the water flux is either monotonically increasing or monotonically decreasing, according to the increase in the driving pressure. This rule also holds for a case in which the water transport results from an osmotic pressure difference. This postulate is supported by numerous studies, such as Oh et al. [23], He et al. [17], and Gaetan et al. [24]. The second postulate concerns the definition of “the ideal semi-permeable membrane.”

**Postulate** **2.** *“The ideal semi-permeable membrane” refers to a membrane that only allows solvent molecules in a solution to pass through and that perfectly rejects solute molecules in a solution.* (P.2)

Hereafter, the ideal semi-permeable membrane described in postulate (P.2) is referred to as “the ideal membrane” for the sake of convenience. The ideal membrane has features described in the following postulates.

**Postulate** **3.** *The performance, such as the magnitude of water transport and solute rejection rate, of the ideal membrane is always higher than that of the actual membranes.* (P.3)

**Postulate** **4.** *The ideal membrane is not realizable.* (P.4)

Note that the performance of the actual membranes mentioned in postulate (P.3) cannot equal that of the ideal membrane because postulate (P.4) states that the ideal membrane is not realizable. In addition to these postulates regarding the ideal membrane, a definition of the ideal membrane-based desalting system is necessary. The following postulate defines actual membrane-based desalting systems and is required for defining the ideal membrane-based desalting system.

**Postulate** **5.** *The magnitudes of the bulk driving pressures in actual membrane-based desalting systems must be dissipated, and the magnitudes of the effective driving pressures exerted on a semi-permeable membrane are always smaller than those of the bulk driving pressures.* (P.5)

Postulate (P.5) is valid due to the energy conservation rule. This postulate is true because driving pressures are a type of specific energy (see Section 2.2). In contrast to actual systems, where the magnitudes of the bulk driving pressures are not conserved, the ideal membrane-based desalting system always guarantees the conservation of driving pressures.

**Postulate** **6.** *“The ideal membrane-based desalting system” stands for a system in which the ideal membrane is employed and the magnitudes of the bulk driving pressures are conserved.* (P.6)

**Postulate** **7.** *The ideal system is not realizable in practice.* (P.7)

Hereafter, the ideal membrane-based desalting system described in postulates (P.6) and (P.7) is referred to as “the ideal system” for the sake of convenience. Likewise, actual membrane-based desalting systems are referred to as “actual systems.” Note, however, that the ideal system and the ideal membrane are concepts that are distinct from one another. The ideal system guarantees the presence of the ideal membrane. However, the ideal membrane may not belong to the ideal system. That is, the ideal membrane can theoretically exist in the actual system. The difference between these ideal concepts is quantified below.

### 2.2. Redefining the Model for Membrane-Based Desalting Processes

In principle, pressure, including the pressure exerted by fluids, is defined as an amount of mechanical force applied to a given area. This definition is often manipulated into a form that describes the amount of energy in a given volume. Should someone desire to observe a change in pressure, the aforementioned definition can be restated as follows:

(1) $$\Delta P=\;-\frac{dF}{dA}=-\frac{dE}{dV}.$$

Here, ΔP is the change in pressure (unless otherwise noted, it hereafter represents the bulk hydraulic pressure in a membrane-based desalting system); F is the mechanical force; A is the given area; E is the energy; and V is the given volume. To measure the change in pressure, a set of conditions (either F and A or E and V) is required. Meanwhile, the osmotic pressure difference is generally represented as a function with respect to the solute concentrations of the less concentrated solution and the more concentrated solution, shown as follows:

(2) $$\Delta \pi =f\left(C_{h},C_{l}\right).$$

**Figure 1:**
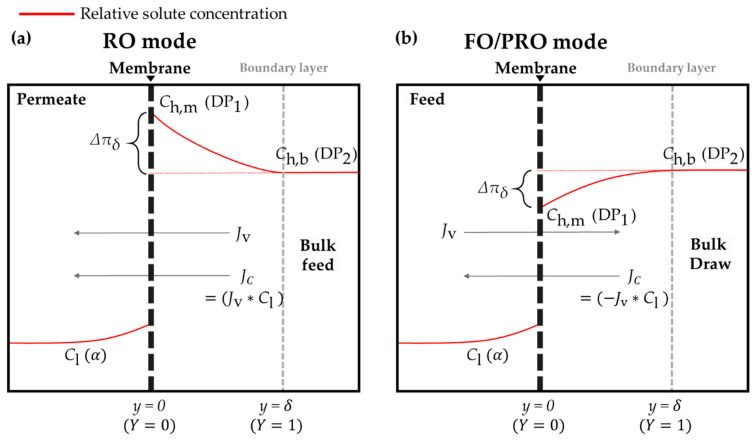
Schematic diagrams illustrating the flow of the water flux ($J_{v}$) and the salt flux ($J_{C}$) in (**a**) the reverse osmosis (RO) mode and (**b**) the forward osmosis (FO) and pressure-retarded osmosis (PRO) modes. Note that the minus sign is appended to the salt flux in FO/PRO modes due to the opposite directions of the water flux and the salt flux. The red curves in each figure represent the relative change of the solute concentration. The larger the solute concentration, the higher the red curve is placed. In the RO mode, the solute concentration at the surface of a membrane in the more concentrated side $\left(C_{h,m}\right)$ is higher than the bulk solute concentration in the more concentrated side $\left(C_{h,b}\right)$. On the other hand, $C_{h,b}$ is higher than $C_{h,m}$ in the FO/PRO modes. Such a difference in the distribution of the solute concentration is what distinguishes the two main types of membrane-based desalting processes.

Here, Δπ is the bulk osmotic pressure difference between the less concentrated solution and the more concentrated solution; $C_{h}$ is the solute concentration of the more concentrated solution; and $C_{l}$ is the solute concentration of the less concentrated solution. In FO and PRO, the more concentrated solution and the less concentrated solution are denoted by “draw solution” and “feed solution,” respectively (see Figure 1). On the other hand, in RO, these are denoted by “feed solution” and “permeate,” respectively. In FO and PRO, water molecules shift from the feed side to the draw side because water transport is mainly driven by the osmotic pressure difference between the feed solution and the draw solution. By contrast, in RO, hydraulic pressure plays a role in forcing water molecules in the feed solution to move to the permeate side. That is, water transport directions of FO/PRO and RO modes are opposites (see Figure 1).

As mentioned before, no equation or model can exactly depict the distribution of osmotic pressure. Hence, Equation (2) might be the most appropriate way of denoting the function of osmotic pressure. Nonetheless, although the exact equation for the value of osmotic pressure is unknown, Δπ can be interpreted as the change of the specific energy caused by the difference between $C_{l}$ and $C_{h}$ in a given solution volume, according to the definition given in Equation (1). Thus, postulate (P.5) can be considered to be correct. Conventional models of membrane-based desalting processes were devised to represent transport phenomena with a driving force defined by the solute concentration difference between $C_{l}$ and $C_{h}$. In order to redefine the conventional transport model, as the current study aims to do, the following relation is used as a starting point:

(3) $$D_{i}^{*}\frac{dX_{i}}{dy}=J_{v}\left(X_{i}-X_{\alpha }\right).$$

Here, Equation (3) provides a general version of the differential equation that is utilized to derive models for membrane-based desalting systems, which is usually harnessed in the form of a local balance of solute transport for an RO process [25,26,27]. Generalizing the differential equations regarding the membrane-based desalting systems, like Equation (3), can be justified since the transport equations in membrane processes are established by the principle that the driving force is produced by a product of the diffusivity coefficient and chemical potential gradient [28]. In Equation (3), $X_{i}$ is the transported variable of the system; $X_{\alpha }$ is a constant value of the transported variable on the less concentrated side; $D_{i}^{*}$ is the diffusivity defined under the condition of the transported variable $X_{i}$; y is the horizontal distance from a given membrane; and $J_{v}$ is the water flux across the membrane. Note that $X_{\alpha }$ is assumed to be a constant because its value is significantly lower than that of the transported variable on the more concentrated side in general. Equations (4a) and (4b), shown below, assign these terms to the case of the conventional model and to the case of the redefined model, respectively.

(4a) $$\{\begin{array}{c} D_{C}^{*}=D\\ X_{c}=C \end{array}\;\mathrm{and}$$

(4b) $$\{\begin{array}{c} D_{DP}^{*}=T_{p}\\ X_{DP}=DP \end{array}.$$

Here, C and DP represent solute concentration and driving pressure, respectively, which are the transported variables in membrane-based desalting systems; D and $T_{p}$ stand for diffusivities in the cases in which the driving forces are the solute concentration difference and the driving pressure difference, respectively. Conventional transport models for membrane-based desalting systems can be obtained if the differential equation shown in Equation (3) is solved after taking D and C to stand for diffusivity and transported variables, respectively. A procedure to solve this differential equation is identical to finding a solution for the well-known Fick’s law that describes the transport of diffusion. [29] Since C and DP are interchangeable because of the equations of state, Equation (3) can be utilized to find equations with respect to DP as well (see Appendixe A and Appendix B for detailed derivations). Despite the different notations, the units for D and $T_{p}$ are alike (m^2^/s). This unit rule is widely accepted, even for other types of diffusivities, such as heat transfer diffusivity and momentum transfer diffusivity. Such a coincidence leads to the advent of the same type of mass transfer coefficient—a key parameter in determining the characteristics of membrane-based desalting systems.

(5) $$\{\begin{array}{c} k_{C}=\frac{D_{C}^{*}}{\delta }=\frac{D}{\delta }\\ k_{DP}=\frac{D_{DP}^{*}}{\delta }=\frac{T_{p}}{\delta } \end{array}.$$

Here, k represents the mass transfer coefficient for the boundary layer of the more concentrated side in membrane-based desalting systems and δ stands for the thickness of a boundary layer in the vicinity of a membrane (see Figure 1). Inside the boundary layer, the solute concentration of the more concentrated solution slightly deviates from that of the bulk more concentrated solution. This phenomenon is called “concentration polarization.” The tendency toward concentration polarization in the boundary layer of the more concentrated side differs in accordance with the type of membrane-based desalting process. In RO, the solute concentration inside the boundary layer is higher than the solute concentration of the bulk feed solution because the hydraulic pressure forces the RO feed solution to shift forward to the membrane [30,31]. While the bulk RO feed solution attempts to get across the membrane, only the solvents in the solution permeate through the membrane; the solutes in the solution are left behind. Thus, the solutes in the RO feed solution are gradually accumulated within the region of the boundary layer. The degree of the solute concentration accumulation can vary as the solute concentration of the bulk feed changes. That is, RO using seawater as the bulk feed should show a higher solute concentration accumulation in the boundary layer than RO using brackish water as the bulk feed. Nonetheless, regardless of the bulk feed type, it was already clarified that the solute concentration of bulk feed in RO is always smaller than that of the boundary layer [32]. Therefore, the fact that the solute concentration of the bulk feed is smaller than that of the boundary layer can generally be applied in RO. However, to avoid confusion among readers, $C_{h}$ and $C_{l}$ are assumed to be 35,000 ppm and 500 ppm, respectively, in the current study. Each value represents the global average salinity of seawater and the maximum total dissolved solid of potable water recommended by the World Health Organization [33].

Something that should be noted is that the hydraulic pressure is not the only factor making the solute concentration of the boundary layer higher than that of the bulk side in RO. For example, the velocity profile of the water flux, which takes place around the membrane surface, also contributes to increasing the solute concentration of the boundary layer [29]. Furthermore, foulants attached to a membrane due to long-term operation can accelerate the accumulation of the solute concentration in the boundary layer because the unwanted substances prevent the solutes from getting across the membrane [34]. Therefore, in actual RO processes, the presence of the other external factors should be considered as well to evaluate the loss of the hydraulic pressure. To deal with those malign factors, researchers have attempted a variety of optimization strategies such as applying optimal membrane spacers to RO processes [35,36,37]. However, the effects of the other harmful factors on the solute concentration are not taken into account in the current study because the objective of this paper is to establish a relation between the osmotic pressure and hydraulic pressure.

In contrast to RO, the solute concentration within the region of the boundary layer in FO and PRO modes is lower than that in the bulk draw solution because the water solvents that pass through the membrane contribute to diluting the concentration [8,38,39]. Such a phenomenon becomes even more obvious if the values of $C_{h}$ and $C_{l}$ are assumed to be as mentioned above. The concentration polarization that occurs in the boundary layer in FO/PRO modes is specifically called the “external dilutive concentration polarization” [4,38]. Taking such tendencies into account, the distribution of the solute concentration within the boundary layer can be described as follows:

(6a) $$\{\begin{array}{c} C=C_{h,m}\;\left(Y=0\right)\\ C=C_{h,b}\;\left(Y=1\right) \end{array}$$

Here, $C_{h,m}$ and $C_{h,b}$ are the solute concentrations of the more concentrated solution at the surface of a membrane and in the bulk region, respectively, and Y is the dimensionless distance from the membrane, which is defined as Y=y/δ [40]. Using the notation of Equation (6a), membrane-based desalting systems can be classified into two types. Systems of the first type, where $C_{h,b}>C_{h,m}$, are usually labeled as either FO or PRO. Systems of the second type, where $C_{h,b}<C_{h,m}$, are usually labeled as RO. Likewise, the distribution of driving pressures can be described as follows:

(6b) $$\{\begin{array}{c} DP=DP_{1}\;\left(Y=0\right)\\ DP=DP_{2}\;\left(Y=1\right) \end{array}$$

Here, $DP_{1}$ and $DP_{2}$ are the driving pressures in membrane-based desalting systems at the surface of a membrane and in the bulk region, respectively. Since the actual driving pressures in these systems are combinations of the bulk hydraulic pressure and the bulk osmotic pressure, the precise denotations of $DP_{1}$ and $DP_{2}$ are unavailable for now. Thus, DPs at each point are simply expressed with subscripts 1 and 2, which are formulated below.

### 2.3. Definitions of Pseudo-Driving Pressures

By incorporating Equation (3) with Equations (4a), (4b), (6a) and (6b), the following two equations for the water flux can be derived:

(7) $$J_{v,C}=-k_{C}\ln\frac{\left(C_{h,m}-C_{l}\right)}{\left(C_{h,b}-C_{l}\right)}\;\mathrm{and}$$

(8) $$J_{v,DP}=-k_{DP}\ln\frac{\left(DP_{1}-\alpha \right)}{\left(DP_{2}-\alpha \right)}.$$

Here, $C_{l}$ and α are the concentration of the less concentrated solution and the arbitrary pressure applied to the less concentrated side, respectively (see Figure 1). That is, $X_{\alpha }=C_{l}$ when the transported variable is the solute concentration, and $X_{\alpha }=\alpha$ when the transported variable is the driving pressure. Equation (7) is obtainable when Equation (3) is solved based on the conditions of Equations (4a) and (6a). This equation is one of the conventional transport models for membrane-based desalting systems, called Brian’s equation [25,26,27]. According to Brian’s equation, the salt flux ($J_{C}$) in the RO mode is estimated as $J_{C}=J_{v}*C_{l}$. In the case of FO/PRO modes, the salt flux ($J_{C}$) in the RO mode should be estimated as $J_{C}=-J_{v}*C_{l}$ because the direction of the salt flux is opposite to that of the water flux. Meanwhile, Equation (8) is newly established in the current study, providing the simplest form of the water flux with respect to the driving pressures that can be derived from Equation (3) (see Appendix A). Although the notations are different, Equations (7) and (8) both represent the water flux in membrane-based desalting systems identically. Accepting that $J_{v,C}=J_{v,DP}$, the following relation comes out based on the logarithm rule:

(9) $$\left\{\frac{\left(C_{h,m}-C_{l}\right)}{\left(C_{h,b}-C_{l}\right)}\right\}={\left\{\frac{\left(DP_{1}-\alpha \right)}{\left(DP_{2}-\alpha \right)}\right\}}^{\frac{k_{DP}}{k_{C}}}.$$

In Equation (9), by allocating the power term, $\frac{k_{DP}}{k_{C}}$, equally to both the denominator and the numerator, new variables for this study can finally be defined.

(10) $$\Delta P_{\mathrm{pse}}\equiv {\left(DP_{1}-\alpha \right)}^{\frac{k_{DP}}{k_{C}}}$$

(11) $$\Delta \pi_{\mathrm{pse}}\equiv {\left(DP_{2}-\alpha \right)}^{\frac{k_{DP}}{k_{C}}}.$$

$\Delta \pi_{\mathrm{pse}}$ of Equation (10) and $\Delta P_{\mathrm{pse}}$ of Equation (11) stand for pseudo-osmotic pressure and pseudo-hydraulic pressure, respectively. The subscript pse stands for pseudo-driving pressures in membrane-based desalting systems. Hereafter, in the current study, $J_{v,C}$ and $J_{v,DP}$ are unified as $J_{v}$ because these notations are accepted as being the same. In addition, due to the definitions of Equations (10) and (11), $k_{DP}$ and $k_{C}$ are also unified as k unless otherwise denoted (see Appendix C). In the end, according to Equations (8), (10), and (11), $J_{v}=-k\ln\left(\frac{\Delta P_{\mathrm{pse}}}{\Delta \pi_{\mathrm{pse}}}\right)$. Each pseudo-driving pressure contributes to determining the direction of $J_{v}$, regardless of the actual magnitudes of the bulk driving pressures. In other words, the direction of $J_{v}$ can be mathematically noted by letting the sign of $J_{v}$ be changed along with $\Delta \pi_{\mathrm{pse}}$ and $\Delta P_{\mathrm{pse}}$. In the current study, a positive value of $J_{v}$ is defined as an indicator of the water flux from a less concentrated side (the left side of the dashed black membrane in Figure 1) to a more concentrated side (the right side of the dashed black membrane in Figure 1). In contrast, a negative value of $J_{v}$ is considered to be an indicator of the water flux that flows from the right side to the left side.

(12) $$\{\begin{array}{c} J_{v}:\mathrm{from}\;\mathrm{left}\;\mathrm{to}\;\mathrm{right}\;(\Delta \pi_{\mathrm{pse}}>\Delta P_{\mathrm{pse}},\;\mathrm{FO}/\mathrm{PRO}\;\mathrm{modes})\\ J_{v}:\mathrm{No}\;\mathrm{flux}\;(\Delta \pi_{\mathrm{pse}}>\Delta P_{\mathrm{pse}})\\ J_{v}:\mathrm{from}\;\mathrm{right}\;\mathrm{to}\;\mathrm{left}\;(\Delta \pi_{\mathrm{pse}}<\Delta P_{\mathrm{pse}},\;\mathrm{RO}\;\mathrm{mode}) \end{array}.$$

Equation (12) shows that the definitions of pseudo-driving pressures are adequate. If a denominator term in Equation (7) is larger than a numerator term in the equation, then $C_{h,b}>C_{h,m}$. Therefore, a given membrane-based desalting system is operated under the condition of FO/PRO modes, implying that the direction of the water flux is from left to right. This result coincides with the fact that the denominator term in $J_{v}=-k\ln\left(\frac{\Delta P_{\mathrm{pse}}}{\Delta \pi_{\mathrm{pse}}}\right)$. is larger than the numerator term. This tendency holds for the RO mode as well. Once again, the criteria given by Equation (12) have nothing to do with the actual driving pressures, such as Δπ and ΔP. To shed light on the relation between the criteria in Equation (12) and the actual driving pressures, the relation given by Equation (9) needs to be scrutinized further. If the numerator and denominator terms on both sides of Equation (9) are connected in parallel, then the following relations can also be obtained:

(13) $$\Delta \pi_{\mathrm{pse}}=a_{\pi }\left(C_{h,b}-C_{l}\right)\;\mathrm{and}$$

(14) $$a_{P}\Delta P_{\mathrm{pse}}=C_{h,m}-C_{l}.$$

Here, $a_{\pi }$ and $a_{P}$ are arbitrary coefficients bridging solute concentrations and pseudo-driving pressures. Due to the fact that hydraulic pressure is not a dependent variable with respect to solute concentrations, $a_{P}$ in Equation (14) is not appended to the concentration terms on the right side. Instead, the hydraulic pressure shoves the solutes in the solution to the adjacent region of the membrane. If Equations (13) and (14) are incorporated, then the following relation ensues:

(15) $$\Delta \pi_{\mathrm{pse}}=a_{\pi }a_{P}\Delta P_{\mathrm{pse}}+a_{\pi }\left(C_{h,b}-C_{h,m}\right).$$

As given in Equation (7), $J_{v,C}=0$ if $C_{h,b}-C_{h,m}=0$. Since Equations (7) and (8) are accepted as identical equations in the current study, $J_{v,DP}=0$ if $J_{v,C}=0$. Then, $\frac{\Delta P_{\mathrm{pse}}}{\Delta \pi_{\mathrm{pse}}}=1$ as $J_{v,DP}$ becomes zero. This feature leads to the following relation:

(16) $$a_{\pi }a_{P}=1.$$

Thus, Equation (15) becomes:

(17) $$\Delta \pi_{\mathrm{pse}}=\Delta P_{\mathrm{pse}}+a_{\pi }\left(C_{h,b}-C_{h,m}\right).$$

A new term given in Equation (17), $a_{\pi }\left(C_{h,b}-C_{h,m}\right)$, needs to be defined as well. Since $a_{\pi }$ is the arbitrary coefficient for the pseudo-osmotic pressure and $C_{h,b}$ and $C_{h,m}$ are the boundary conditions for the boundary layer, this new term can be defined as follows:

(18) $$\Delta \pi_{\delta ,\mathrm{pse}}=a_{\pi }|C_{h,b}-C_{h,m}|.$$

Here, $\Delta \pi_{\delta ,\mathrm{pse}}$ denotes the pseudo-osmotic pressure inside the boundary layer of the more concentrated solution. In the current study, negative pressure is not taken into account. Thus, $\Delta \pi_{\delta ,\mathrm{pse}}$ must be set as a positive number with absolute value bars. Due to the rule of absolute value bars, $\Delta \pi_{\delta ,\mathrm{pse}}=a_{\pi }\left(C_{h,b}-C_{h,m}\right)$ while $C_{h,b}>C_{h,m}$. On the other hand, $\Delta \pi_{\delta ,\mathrm{pse}}=-a_{\pi }\left(C_{h,b}-C_{h,m}\right)=a_{\pi }\left(C_{h,m}-C_{h,b}\right)$ while $C_{h,b}<C_{h,m}$. Therefore, Equation (17) can be restated as follows:

(19) $$\Delta \pi_{\mathrm{pse}}=\Delta P_{\mathrm{pse}}+\Delta \pi_{\delta ,\mathrm{pse}}\;(\mathrm{for}\;\mathrm{FO}\;\mathrm{and}\;\mathrm{PRO})\;\mathrm{and}$$

(20) $$\Delta \pi_{\mathrm{pse}}=\Delta P_{\mathrm{pse}}-\Delta \pi_{\delta ,\mathrm{pse}}\;(\mathrm{for}\;\mathrm{RO}).$$

Please note that $\Delta \pi_{\mathrm{pse}}$ cannot be zero. Systems of interest in the current study are based on an assumption that osmotic pressure exists at any time; this is the definition of membrane-based “desalting” systems. Equations (19) and (20) show that the difference between two pseudo-driving pressures, $\Delta \pi_{\mathrm{pse}}-\Delta P_{\mathrm{pse}}$ (or $\Delta P_{\mathrm{pse}}-\Delta \pi_{\mathrm{pse}}$), is $\Delta \pi_{\delta ,\mathrm{pse}}$. Taking Equations (1) and (2) into consideration, it is evident that the physical implication of $\Delta \pi_{\delta }$ is the amount of energy within the volume of the boundary layer. That is, Equations (19) and (20) can be regarded as energy balances that govern membrane-based desalting systems. In order to set these energy balances more accurately, new coefficients need to be defined.

### 2.4. Similarity Coefficients and the Reflection Coefficient

In this study, new coefficients are harnessed in order to bridge the gap between the measurable driving pressures (i.e., Δπ and ΔP) and the intangible driving pressures (i.e., $\Delta \pi_{\mathrm{pse}}$ and $\Delta P_{\mathrm{pse}}$). The new coefficients are defined as follows:

(21) $$\Delta \pi_{\mathrm{pse}}=S_{\pi }\Delta \pi \;(0<S_{\pi }\leq 1)\;\mathrm{and}$$

(22) $$\Delta P_{\mathrm{pse}}=S_{P}\Delta P\;(0<S_{P}\leq 1).$$

In the same manner, $\Delta \pi_{\delta ,\mathrm{pse}}=S_{\pi }\Delta \pi_{\delta }$. $S_{\pi }$ and $S_{P}$ are similarity coefficients for the pseudo-osmotic pressure and the pseudo-hydraulic pressure, respectively. Similarity coefficients bridge the measurable (real) driving pressures and the intangible (pseudo) driving pressures. That is, a similarity coefficient is analogous to the fugacity coefficient used in chemical engineering thermodynamics [41]. Since the value of pseudo-driving pressure cannot surpass that of real driving pressure, the similarity coefficient always ranges from zero to one. In this regard, the physical implication of the similarity coefficient can be interpreted as the degree of energy loss from the state of pure driving pressure. That is, the postulation of postulate (P.5), which represents the energy conservation rule, is embodied by similarity coefficients, the values of which range from zero to one.

In order to represent the tendencies of membrane-based desalting systems, another coefficient needs to be utilized from past studies. This is the reflection coefficient, which is a system parameter defined in the Kedem–Katchalsky model [42,43,44] and the Spiegler–Kedem model [45,46,47]. It is represented below.

(23) $$\sigma ={\left(\frac{\Delta P}{\Delta \pi }\right)}_{J_{v}=0}.$$

Here, σ denotes the reflection coefficient. If σ=1, then a given membrane is considered to be an ideal one that can perfectly reject solutes and only allow solvents to flow through. If 0<σ<1, then a given membrane is classified as being an actual type, capable of preventing solutes from getting across it, to some extent, though it cannot do so perfectly. When σ=0, a given membrane cannot reject the solutes at all. Here, note that the reflection coefficient is just a phenomenological parameter. That is, the reflection coefficient cannot provide a specific value regarding the performance of a membrane. By adding Equations (21)–(23), the reflection coefficient can be rewritten as follows:

(24) $$\sigma ={\left(\frac{\Delta P}{\Delta \pi }\right)}_{J_{v}=0}={\left(\frac{S_{\pi }\Delta P_{\mathrm{pse}}}{S_{P}\Delta \pi_{\mathrm{pse}}}\right)}_{J_{v}=0}.$$

When $J_{v}=0$, then $\frac{\Delta P_{\mathrm{pse}}}{\Delta \pi_{\mathrm{pse}}}=1$. Therefore, $\sigma =\frac{S_{\pi }}{S_{P}}$ when $J_{v}=0$. Consequently, the criteria for the reflection coefficient represented above can be rewritten as follows when $J_{v}=0$:

(25) $$\{\begin{array}{c} S_{\pi }=S_{P}\;\left(\sigma =1\right)\\ 0<S_{\pi }<S_{P}\;(0<\sigma <1)\\ S_{\pi }=0\;(\sigma =0) \end{array}.$$

With Equation (25), the difference between the ideal membrane and the ideal system is clarified. Since the ideal system always guarantees conservation of the bulk driving pressures, $S_{\pi }=S_{P}=1$ in the ideal system at any time. In the ideal system, $J_{v}=0$ only when Δπ=ΔP. Furthermore, in the ideal system, the amount of increase (or decrease) in ΔP is equally converted into the decrease (or increase) in Δπ, and vice versa. That is, a relation of $S_{\pi }=S_{P}=1$ represents that a given system comprises equivalent Δπ and ΔP, which people usually imagine. On the other hand, the ideal membrane does not imply conservation of the bulk driving pressures. Therefore, the values of $S_{\pi }$ and $S_{P}$ need not be equal, except for the moment $J_{v}=0$ in a system in which only the ideal membrane is employed. Furthermore, the values of $S_{\pi }$ and $S_{P}$ do not need to be 1 even at the moment $J_{v}=0$. For example, when $J_{v}=0$, a relation of $S_{\pi }=S_{P}=\frac{1}{2}$ suffices to describe the ideal membrane condition shown in Equation (25). Therefore, the ideal membrane and the ideal system are totally different concepts except for the fact that the water flux ceases when Δπ=ΔP. In the current study, when discussing differences between ideal and actual systems, it is assumed that the ideal membrane is always employed in the ideal system.

### 2.5. A Relation between Osmotic Pressure and Hydraulic Pressure

When similarity coefficients are defined, then relations between the bulk driving pressures can be expressed using them. Equations (19) and (20) can be rearranged as Equations (26) and (27), respectively, when incorporated with Equations (21) and (22).

(26) $$\Delta \pi =\frac{S_{P}}{S_{\pi }}\Delta P+\frac{\Delta \pi_{\delta ,\mathrm{pse}}}{S_{\pi }}=\frac{S_{P}}{S_{\pi }}\Delta P+\Delta \pi_{\delta }\;(\mathrm{FO}\;\mathrm{and}\;\mathrm{PRO})\;\mathrm{and}$$

(27) $$\Delta \pi =\frac{S_{P}}{S_{\pi }}\Delta P-\frac{\Delta \pi_{\delta ,\mathrm{pse}}}{S_{\pi }}=\frac{S_{P}}{S_{\pi }}\Delta P-\Delta \pi_{\delta }\;(\mathrm{RO}).$$

As shown in Equations (26) and (27), the ratio of similarity coefficients plays a key role in finding the relation between driving pressures of membrane-based desalting systems. Hence, it is worth investigating how $\frac{S_{P}}{S_{\pi }}$ changes in accordance with the configurations of the systems.

By defining similarity coefficients, another important relation between osmotic pressure and hydraulic pressure can be derived from Equation (14). As given by Equation (16), $\frac{1}{a_{\pi }}=a_{P}$. Thus, Equation (14) can be rewritten as follows:

(28) $$\Delta P_{\mathrm{pse}}=a_{\pi }\left(C_{h,m}-C_{l}\right).$$

As was done in Equation (18), the right-side term in Equation (28) can additionally be denoted. Since $C_{h,m}$ is always larger than $C_{l}$, the right-side term in Equation (28) can be defined as:

(29) $$\Delta \pi_{m,\mathrm{pse}}=a_{\pi }\left(C_{h,m}-C_{l}\right).$$

Here, $\Delta \pi_{m,\mathrm{pse}}$ represents the pseudo-osmotic pressure confined to the region of membrane inside. By combining Equations (21), (22), (28), and (29), the following relation is obtained:

(30) $$\frac{\Delta P}{\Delta \pi_{m}}=\frac{S_{\pi }}{S_{P}}.$$

The relation represented by Equation (30) is always applicable to all types of membrane-based desalting systems. By substituting the term $\frac{S_{P}}{S_{\pi }}$ of $J_{v}=-k\ln\left(\frac{S_{P}\Delta P}{S_{\pi }\Delta \pi }\right)$ with Equation (30), an equation for the water flux can be newly obtained.

(31) $$J_{v}=-k\ln\left(\frac{\Delta \pi_{m}}{\Delta \pi }\right).$$

Thus, Equation (31) is the water flux equation that is applicable to all types of membrane-based desalting systems and at any time. In particular, Equation (31) is useful when ΔP≈0, which is a conventional operational condition for FO. By calculating $\Delta \pi_{m}$ numerically, Equation (31) can be found.

## 3. Results and Discussions

In the current section, the basic theory devised above is expanded. The results corresponding to the expanded theory are subsequently analyzed and the implications of this analysis are described.

### 3.1. A Constraint for the Monotonic Functions by the Similarity Coefficient Ratio

In the previous section, similarity coefficients are defined in order to understand the gap between the bulk driving pressures and the pseudo-driving pressures. Although the equations relating to similarity coefficients are not known, it is possible to find the constraint required for determining the aforementioned monotonic functions. In the current study, as assumed by postulate (P.1), the plots with respect to the water flux are either monotonically increasing or monotonically decreasing. In addition to the postulate, given in Equation (12), recall that $\frac{\Delta P_{\mathrm{pse}}}{\Delta \pi_{\mathrm{pse}}}=\frac{S_{P}}{S_{\pi }}\frac{\Delta P}{\Delta \pi }\leq 1$ in the FO and PRO modes and $\frac{\Delta P_{\mathrm{pse}}}{\Delta \pi_{\mathrm{pse}}}=\frac{S_{P}}{S_{\pi }}\frac{\Delta P}{\Delta \pi }>1$ in the RO mode. In this study, the sign of the water flux is assumed to be positive in the FO and PRO modes and negative in the RO mode, and the change of $\frac{S_{P}}{S_{\pi }}\frac{\Delta P}{\Delta \pi }$, according to $\frac{\Delta P}{\Delta \pi }$, must be larger than zero. That is, the following inequality is always valid for membrane-based desalting systems:

(32) $$\frac{d\left(uv\right)}{dv}\geq 0.$$

Here, $u=\frac{S_{P}}{S_{\pi }}$ and $v=\frac{\Delta P}{\Delta \pi }$. According to the total derivative rule, Equation (32) becomes:

(33) $$\frac{d\left(uv\right)}{dv}=\frac{\partial \left(uv\right)}{\partial u}\frac{\partial u}{\partial v}+\frac{\partial \left(uv\right)}{\partial v}\frac{\partial v}{\partial v}\geq 0.$$

Consequently, the following inequality is revealed after Equation (33) is appropriately rearranged:

(34) $$\frac{\partial u}{\partial v}\geq -\frac{u}{v}↔\frac{\partial \left(\frac{S_{P}}{S_{\pi }}\right)}{\partial \left(\frac{\Delta P}{\Delta \pi }\right)}\geq -\frac{\frac{S_{P}}{S_{\pi }}}{\frac{\Delta P}{\Delta \pi }}.$$

Equation (34) is the one and only constraint for fixing the plots with respect to the water flux as monotonic functions. It can be rewritten as follows by incorporating Equations (26) and (27):

(35) $$\frac{\partial \left(\frac{S_{P}}{S_{\pi }}\right)}{\partial \left(\frac{\Delta P}{\Delta \pi }\right)}\geq -\frac{\frac{S_{P}}{S_{\pi }}}{\frac{\Delta P}{\Delta \pi }}=-\frac{\Delta \pi \Delta \pi_{m}}{\Delta P^{2}}-\frac{\Delta \pi^{2}-\Delta \pi \Delta \pi_{\delta }}{\Delta P^{2}}\;(\mathrm{FO}\;\mathrm{and}\;\mathrm{PRO})\;\mathrm{and}$$

(36) $$\frac{\partial \left(\frac{S_{P}}{S_{\pi }}\right)}{\partial \left(\frac{\Delta P}{\Delta \pi }\right)}\geq -\frac{\frac{S_{P}}{S_{\pi }}}{\frac{\Delta P}{\Delta \pi }}=-\frac{\Delta \pi \Delta \pi_{m}}{\Delta P^{2}}=-\frac{\Delta \pi^{2}+\Delta \pi \Delta \pi_{\delta }}{\Delta P^{2}}\;(\mathrm{RO}).$$

Equations (35) and (36) are critical because these inequalities show the constraints of “coupled” similarity coefficients. In other words, one similarity coefficient cannot solely have a critical impact on membrane-based desalting systems. This is the reason why the similarity coefficients are collectively considered as given in Equation (25). Only when both coefficients are taken into consideration are the overall optimization works of membrane-based desalting processes realizable. Equations (31) and (32) are intriguing in that they have no limit to the positive value of $\frac{\partial \left(\frac{S_{P}}{S_{\pi }}\right)}{\partial \left(\frac{\Delta P}{\Delta \pi }\right)}$. If the conditions of Equations (35) and (36) are fulfilled, then the plots of the water flux always change monotonically.

### 3.2. Verification of the Relation between the Driving Pressures in FO and PRO Modes

In FO and PRO modes, a constraint with respect to the ideality of a membrane should be derived in order to investigate the tendencies of the corresponding processes. According to postulate (P.3), the magnitude of water transport conducted with the ideal membrane must be higher than that conducted with the actual membranes. This can be expressed as follows:

(37) $$\left|J_{v,\mathrm{ideal}}\right|-\left|J_{v,\mathrm{actual}}\right|>0.$$

Here, the subscripts “ideal” and “actual” indicate the ideal system and the actual system, respectively. Since the ideal system guarantees the presence of the ideal membrane, postulate (P.3) also describes the ideal system. In the ideal system, Δπ>ΔP in FO and PRO modes, as postulate (P.6) states ($∵S_{\pi }=S_{P}=1$). When an actual membrane is employed, then $\Delta \pi_{\mathrm{pse}}>\Delta P_{\mathrm{pse}}$ in FO and PRO modes. Thus, according to Equation (8), $\left|J_{v,\mathrm{ideal}}\right|=-k\ln\left(\frac{\Delta P}{\Delta \pi }\right)$. On the other hand, $\left|J_{v,\mathrm{actual}}\right|=-k\ln\left(\frac{\Delta P_{\mathrm{pse}}}{\Delta \pi_{\mathrm{pse}}}\right)=-k\ln\left(\frac{S_{P}}{S_{\pi }}\frac{\Delta P}{\Delta \pi }\right)$. Therefore, Equation (33) becomes:

(38) $$k\ln\left(\frac{\Delta P}{\Delta \pi }\right)-k\ln\left(\frac{S_{P}}{S_{\pi }}\frac{\Delta P}{\Delta \pi }\right)=k\ln\left(\frac{S_{\pi }}{S_{P}}\right)<0.$$

Since k is always larger than zero, $\frac{S_{\pi }}{S_{P}}$ must be lower than one, according to Equation (38). That is, $S_{\pi }<S_{P}$ in FO and PRO modes. That is, according to Equations (25), (26), and (38), $\frac{S_{P}}{S_{\pi }}$ is always larger than one in FO and PRO modes. Consequently, Δπ is larger than ΔP, as long as $\Delta \pi_{\mathrm{pse}}>\Delta P_{\mathrm{pse}}$. In other words, $\frac{\Delta P}{\Delta \pi }$ is always lower than one if water molecules transport from the less concentrated side to the more concentrated side. Such a result implies that the overall transport of FO/PRO modes occurs because of the relation $\Delta \pi_{m}>\Delta P$ ($∵\frac{\Delta P}{\Delta \pi_{m}}=\frac{S_{\pi }}{S_{P}}$). Furthermore, Equation (38) also means that the value of the bulk osmotic pressure difference can never be equal to that of the bulk hydraulic pressure in FO and PRO modes, with the exception being when ΔP=Δπ=0.

**Figure 2:**
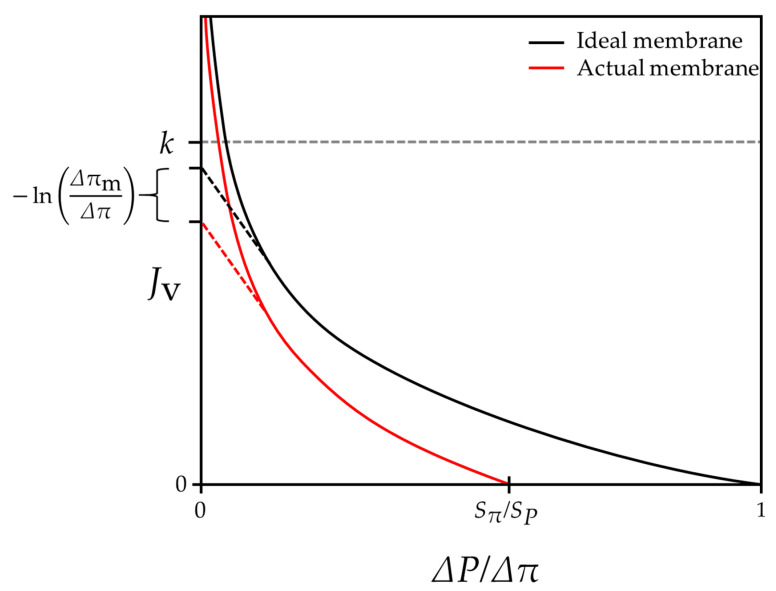
A schematic plot with respect to the relationship between the ratio of the bulk driving pressures $\left(\frac{\Delta P}{\Delta \pi }\right)$ and the value of the water flux $\left(J_{v}\right)$ in FO and PRO modes. A presumed water flux limit in FO and PRO is marked with a dashed gray line. When ΔP=0 (i.e., the FO mode), the water flux must be calculated with $J_{v}=-k\ln\left(\frac{\Delta \pi_{m}}{\Delta \pi }\right)$. On the other hand, the water flux in the PRO mode can be determined with the equation $J_{v}=-k\ln\left(\frac{\Delta P_{\mathrm{pse}}}{\Delta \pi_{\mathrm{pse}}}\right)$. The dashed red line indicates the change of $J_{v}$ with the implementation of an actual membrane and the practical change of $J_{v}$ when ΔP→0. On the other hand, the solid red curve indicates the change of $J_{v}$ when the implementation of an actual membrane is taken into account but the practical change of $J_{v}$ is not. That is, the solid red line does not consider the value of $J_{v}$ when moment ΔP=0. The black curves indicate the changes of $J_{v}$ when an ideal membrane is employed. The difference between the solid and dashed black lines is the same as the difference between the solid and dashed red lines.

In addition, such a result implies that $J_{v}$ by the actual membrane becomes zero at a point that is distant from the point at which $\frac{\Delta P}{\Delta \pi }=1$. According to Equation (8), $J_{v,DP}=0$ when $\frac{\Delta P}{\Delta \pi }=\frac{S_{\pi }}{S_{P}}$ and the value of $\frac{S_{\pi }}{S_{P}}$ is less than one, as mentioned above. As a result, the overall tendency of $J_{v}$ in FO/PRO modes, along with the change of $\frac{\Delta P}{\Delta \pi }$, is represented in Figure 2. Other than the fact that $J_{v}=0$ when $\frac{\Delta P}{\Delta \pi }=\frac{S_{\pi }}{S_{P}}$, more critical implications are hidden in this figure. As $\frac{\Delta P}{\Delta \pi }\to 0$, $J_{v}=-k\ln\left(\frac{S_{P}\Delta P}{S_{\pi }\Delta \pi }\right)$ cannot be applicable because its value goes to infinity. In reality, the value of $J_{v}$ is not infinite, even when ΔP=0, which means that Equation (31) must be used instead when ΔP→0. Such a result implies that the water flux of the FO mode is unpredictable, unless situations inside a membrane are taken into account.

Another important point hidden in Figure 2 is that the presumable water flux limit can exist when it comes to practical FO and PRO modes. Recently, FO and PRO researchers who are trying to improve process performances have begun to slowly recognize that there is a water flux limit regardless of membrane performances [48,49]. Numerous reasons may cause this water flux limit of FO and one of the factors could be attributed to the fact that the value of the water flux does not exceed the value of the mass transfer coefficient of the draw side—namely, k. Although it has not been theoretically proven before, the value of $\frac{J_{v}}{k}$ that is reported in all FO and PRO research is always smaller than one, even in recent studies (see Table 1). Such a tendency can be justified by the fact that $\frac{\Delta \pi_{\delta }}{\Delta \pi }\ll 1$ with the practical membranes and $J_{v}=-k\ln\left(\frac{S_{P}\Delta P}{S_{\pi }\Delta \pi }\right)=-k\ln\left(1-\frac{\Delta \pi_{\delta }}{\Delta \pi }\right)$ for FO and PRO modes. The value of $-\ln\left(1-\frac{\Delta \pi_{\delta }}{\Delta \pi }\right)$ is less than one as long as $\frac{\Delta \pi_{\delta }}{\Delta \pi }$ is smaller than 0.63. Physically, $\frac{\Delta \pi_{\delta }}{\Delta \pi }$ implies the degree of dilutive external concentration polarization (dECP) that takes place on the draw sides of FO and PRO processes. Although the importance of dECP has recently become more emphasized, it is very challenging for the value of $\frac{\Delta \pi_{\delta }}{\Delta \pi }$ to exceed 0.63, considering the osmotic pressure loss that happens in the rest of the regions of a membrane. In particular, it is widely admitted that the internal concentration polarization that occurs in the support layer of a membrane is more influential for a system than dECP [38,50,51]. Therefore, it is acceptable that $J_{v}$ cannot exceed the value of k unless an extremely severe dECP is assumed.

**Figure 3:**
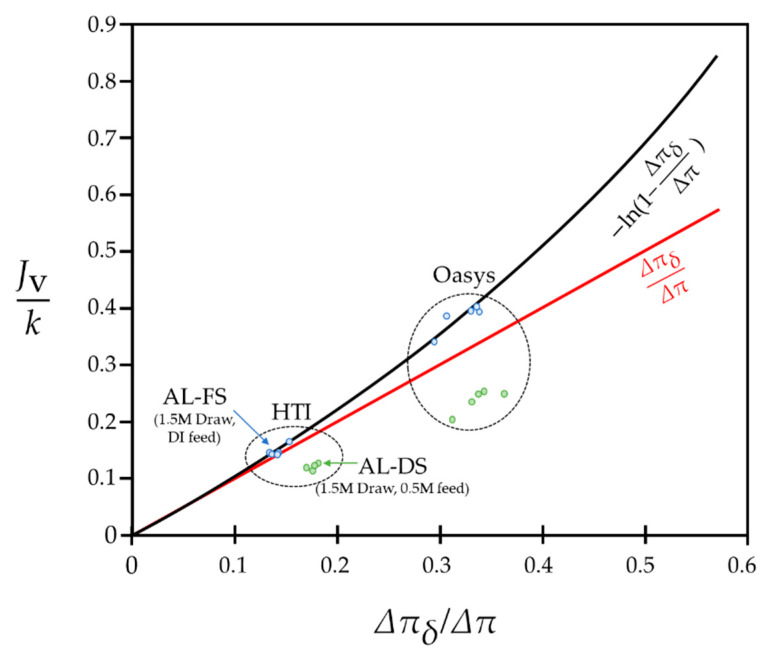
A figure representing the values of $\frac{J_{v}}{k}$, according to $\frac{\Delta \pi_{\delta }}{\Delta \pi }$, using the experimental data from a previous study [50]. In this previous study, the authors used two other FO/PRO membranes that were manufactured by HTI and Oasys. The FO experiment was conducted with the condition of $C_{h,b}=1.5M,\;C_{l,b}=0M$, while the PRO experiment was conducted with $C_{h,b}=1.5M,\;C_{l,b}=0.5M$. The temperature of both experiments was fixed at 20 °C. The straight red line in the figure indicates the approximation of $-\ln\left(1-\frac{\Delta \pi_{\delta }}{\Delta \pi }\right)$, which is applicable when $\frac{\Delta \pi_{\delta }}{\Delta \pi }$ is small enough.

Figure 3 represents the values of $\frac{J_{v}}{k}$, according to $\frac{\Delta \pi_{\delta }}{\Delta \pi }$, using the experimental data from a previous study [50]. In this previous study, the authors used two other FO/PRO membranes that were manufactured by different membrane vendors: Hydration Technology Innovations (HTI) and Oasys. The authors of the study controlled the value of k by varying the crossflow velocity of a channel and found the values of $\frac{\Delta \pi_{\delta }}{\Delta \pi }$ accordingly. The FO experiment was conducted with the condition of $C_{h,b}=1.5M,\;C_{l,b}=0M$, while the PRO experiment was conducted with $C_{h,b}=1.5M,\;C_{l,b}=0.5M$. The temperature of both experiments was fixed at 20 °C.

As clearly shown in Figure 3, the performances of the two types of membranes are highly different. Despite this difference in terms of performances, the experimental data follow the plot of $\frac{J_{v}}{k}=-\ln\left(1-\frac{\Delta \pi_{\delta }}{\Delta \pi }\right)$ comparatively well. Accepting that the relation of $\frac{J_{v}}{k}=-\ln\left(1-\frac{\Delta \pi_{\delta }}{\Delta \pi }\right)$ can be applicable for both FO and PRO modes, regardless of membrane performances, Figure 3 strengthens the possibility of the water flux limit in FO and PRO processes. In the figure, the values of $\frac{J_{v}}{k}$ are less than 0.5 for both membranes. That is, a much higher osmotic pressure loss is required at the membrane interface for a system to make $\frac{J_{v}}{k}$ higher than one. Even when $\frac{\Delta \pi_{\delta }}{\Delta \pi }=0.43$, which is the highest value that was obtained when the temperature was at 40 °C, $-\ln\left(1-\frac{\Delta \pi_{\delta }}{\Delta \pi }\right)$ becomes only 0.562. That is, the mass transfer coefficient on the more concentrated sides in FO and PRO modes can tentatively be considered to be the water flux limit. (The experimental data presented in Figure 3 were used after obtaining appropriate permission for reuse.)

Apart from the aforementioned mathematical proof, the fact that Δπ is always smaller than ΔP in FO and PRO modes can be demonstrated in another way. When $J_{v}\approx 0$, the following relation is valid:

(39) $$\frac{\Delta P_{\mathrm{pse}}}{\Delta \pi_{\mathrm{pse}}}\approx 1=\frac{S_{P}}{S_{\pi }}\frac{\Delta P}{\Delta \pi }.$$

Furthermore, since $\Delta \pi_{\mathrm{pse}}>\Delta P_{\mathrm{pse}}$, the difference between Δπ and ΔP can be represented with Equation (26) as follows:

(40) $$\Delta \pi -\Delta P=\left(\frac{S_{P}}{S_{\pi }}-1\right)\Delta P+\Delta \pi_{\delta }.$$

According to Equation (39), $\Delta P=\frac{S_{\pi }}{S_{P}}\Delta \pi$. If ΔP in Equation (40) is substituted with $\frac{S_{\pi }}{S_{P}}\Delta \pi$, the difference between Δπ and ΔP can be rewritten as follows:

(41) $$\Delta \pi -\Delta P=\left(\frac{S_{P}}{S_{\pi }}-1\right)\frac{S_{\pi }}{S_{P}}\Delta \pi +\Delta \pi_{\delta }=\left(1-\frac{S_{\pi }}{S_{P}}\right)\Delta \pi +\Delta \pi_{\delta }.$$

Recall that $\frac{S_{\pi }}{S_{P}}=\sigma$ when $\frac{\Delta P_{\mathrm{pse}}}{\Delta \pi_{\mathrm{pse}}}=1$. In addition, $\Delta \pi_{\delta }\to 0$ as $J_{v}\to 0$ since $|C_{h,b}-C_{h,m}|$ approaches zero according to Equation (7). Hence, Equation (41) can be restated as follows:

(42) Δπ−ΔP=(1−σ)Δπ.

**Figure 4:**
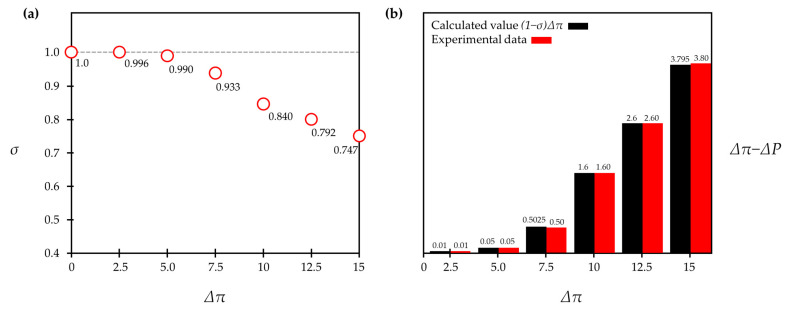
The change of (**a**) the reflection coefficient (σ) and (**b**) the bulk driving pressure difference (Δπ−ΔP ), according to the bulk osmotic pressure difference (Δπ ). The calculated values of (1−σ)π in (**b**) are based on Equation (37) and the experimental data in (**a**,**b**) were adapted with permission from [57]. Copyright 2018, American Chemical Society.

If $J_{v}$ is small enough to approximate the value of $\frac{\Delta P_{\mathrm{pse}}}{\Delta \pi_{\mathrm{pse}}}$ as one, but the value of $J_{v}$ is not zero, then $\Delta \pi_{\delta }$ may not be canceled out. According to other previous studies [57], σ continually decreases as Δπ increases. Therefore, as Equation (42) implies, the difference between Δπ and ΔP gets larger as Δπ increases. Figure 4a displays the experimental data of σ, showing that it continually decreases in accordance with the changes in Δπ [46,57]. By taking this declining tendency of σ into account, the value of Δπ−ΔP can be calculated with Equation (42), and the calculated values of Δπ−ΔP perfectly match the experimental results shown in Figure 4b.

In fact, Equation (42) can also be derived from other thermodynamic models, such as the Kedem–Katchalsky model and the Spiegler–Kedem model. However, these models set Δπ and ΔP as separate variables and do not connect the two directly. By contrast, this study derives Δπ−ΔP by relating these two driving pressures and additionally shows how the difference between Δπ and ΔP can vary when $J_{v}$ is not exactly equal to zero (i.e., $J_{v}\to 0$ but $J_{v}\neq 0$).

The most salutary lesson obtainable from Equations (26), (27), and (39) to (42) is that the similarity coefficient ratio, $\frac{S_{P}}{S_{\pi }}$, is a key factor in determining the performance of membrane-based desalting systems. Conventionally, Δπ−ΔP is set as the net driving pressure. Even when determining the value of Δπ−ΔP, the value of $\frac{S_{P}}{S_{\pi }}$ is important because whether $\left(1-\frac{S_{\pi }}{S_{P}}\right)$, in Equation (41), is positive or negative contributes to determining the sign of Δπ−ΔP. However, as proven earlier, a situation in which $\left(1-\frac{S_{\pi }}{S_{P}}\right)$ is negative does not occur in FO/PRO modes because $\frac{S_{P}}{S_{\pi }}$ is always larger than one. Now, recall the physical implications of each similarity coefficient. As mentioned above, the values of similarity coefficients represent the degree of “energy loss from the bulk driving pressures.” The higher the value of a similarity coefficient, the lower the energy loss from a bulk driving pressure. Thus, the amount of Δπ energy loss is always larger than that of ΔP in FO/PRO modes. Meanwhile, according to Equations (8) and (25), the value of $\frac{S_{P}}{S_{\pi }}$ should be as close to one as possible in order to exhibit the best performance in actual membrane-based desalting systems. Hence, the value of $\frac{S_{P}}{S_{\pi }}$ must be a value larger than one and the difference between $S_{\pi }$ and $S_{P}$ should be kept sufficiently small. In this context, strategies for controlling the values of $S_{\pi }$ and $S_{P}$ should be carefully designed. It is widely known that hydraulic pressure’s energy loss is mainly caused by frictional loss, which is representatively formulated using the Darcy–Weisbach equation [58]. The main contributing factors that cause frictional loss are the operational parameters of membrane-based desalting processes, such as “hydraulic channel height” and “crossflow velocity.” On the other hand, osmotic pressure’s energy loss is attributed to the performance of a membrane [4,39]. If membrane performance is not sufficiently good, then the concentration polarization around the membrane is aggravated. The more severe the concentration polarization is, the further it undermines the effectiveness of osmotic pressure. To alleviate the concentration polarization, membranes with optimal design and spacers should be employed. In short, controlling the value of $S_{\pi }$ is work that involves the optimization of membrane parameters, such as “salt rejection rate”, “salt permeability”, and “optimal spacers”, while controlling the value of $S_{P}$ is work that involves the optimization of operational parameters, such as “hydraulic channel height” and “crossflow velocity.”

Even with a highly optimized membrane, the value of $S_{\pi }$ cannot surpass that of $S_{P}$ in FO/PRO modes because $\frac{S_{P}}{S_{\pi }}$ is always larger than one. That is, in FO/PRO modes, it could be said that the level of membrane optimization is innately limited as long as operational parameters remain the same. Such a difference alludes to what should be done in order to improve the performance of FO/PRO processes. The best status of an FO/PRO process is to produce a water volume that is as large as possible with the smallest energy loss. As explained above, the closer the values of similarity coefficients are to one, the smaller the energy loss. Therefore, both $S_{\pi }$ and $S_{P}$ should be as high as possible. However, solely increasing $S_{\pi }$ has an obvious limit in that the value of $S_{\pi }$ cannot surpass that of $S_{P}$ in FO/PRO modes. Hence, $S_{P}$ needs to be improved before the value of $S_{\pi }$ is augmented. However, solely augmenting $S_{P}$ makes the difference between $S_{\pi }$ and $S_{P}$ greater so that the value of $J_{v}$ decreases. This means that work intended to increase $S_{\pi }$ should be conducted after $S_{P}$ is improved. A series of this logical flow draws the conclusion that only improving either $S_{\pi }$ or $S_{P}$ does not have a big impact on the performance of membrane-based desalting systems. Instead, choosing just one of them might sometimes lead to worse results. As such, both $S_{\pi }$ and $S_{P}$ need to simultaneously be taken into consideration when attempting to enhance the performance of membrane-based desalting processes.

### 3.3. Verification of the Relation between Driving Pressures in the RO Mode

As had been done for FO and PRO modes, the first thing that should be investigated in the RO mode is the inequality, according to postulate (P.3), between $\left|J_{v,\mathrm{ideal}}\right|$ and $\left|J_{v,\mathrm{actual}}\right|$. In the ideal system, Δπ<ΔP in the RO mode, as postulate (P.6) states ($∵S_{\pi }=S_{P}=1$). When an actual membrane is employed, then $\Delta \pi_{\mathrm{pse}}<\Delta P_{\mathrm{pse}}$ in the RO mode. Thus, according to Equation (8), $\left|J_{v,\mathrm{ideal}}\right|=k\ln\left(\frac{\Delta P}{\Delta \pi }\right)$. On the other hand, $\left|J_{v,\mathrm{actual}}\right|=k\ln\left(\frac{\Delta P_{\mathrm{pse}}}{\Delta \pi_{\mathrm{pse}}}\right)=k\ln\left(\frac{S_{P}}{S_{\pi }}\frac{\Delta P}{\Delta \pi }\right)$. Therefore, Equation (37), which was used to derive the constraint for FO and PRO modes, becomes:

(43) $$\left|J_{v,\mathrm{ideal}}\right|-\left|J_{v,\mathrm{actual}}\right|=k\left(\ln\left(\frac{\Delta P}{\Delta \pi }\right)-\ln\left(\frac{S_{P}}{S_{\pi }}\frac{\Delta P}{\Delta \pi }\right)\right)>0.$$

Note that Equation (43) is only valid in the region of $\frac{\Delta P}{\Delta \pi }>1$. Consequently, the tendency of membrane-based desalting systems in the range of $\frac{S_{\pi }}{S_{P}}<\frac{\Delta P}{\Delta \pi }<1$ remains enigmatic for now. Incidentally, Equation (43) shows that $\frac{S_{\pi }}{S_{P}}>1$ in the RO mode. That is, $S_{P}<S_{\pi }$ in the RO mode, while $S_{P}>S_{\pi }$ in FO and PRO modes. Thus, inequality between similarity coefficients can also be an indicator of membrane-based desalting systems. Such differences in the inequalities of the similarity coefficients lead to important lessons, such as those that were obtained during the FO/PRO discussion. First, such a result implies that the overall transport of RO modes occurs because of the relation $\Delta \pi_{m}<\Delta P$ ($∵\frac{\Delta P}{\Delta \pi_{m}}=\frac{S_{\pi }}{S_{P}}$). Furthermore, as discussed above, in FO/PRO modes, the amount Δπ of energy loss is always larger than that of ΔP in FO/PRO modes. However, in RO mode, the amount of energy loss of ΔP is always larger than that of Δπ because $S_{P}<S_{\pi }$. The different sequence for optimization work suggests that distinct strategies are required for each membrane-based desalting system. For a detailed explanation of optimization work, see Section 3.5, which focuses on optimization strategies.

Unfortunately, in the RO mode, a dilemma relating to the preceding postulates and criteria is presented. According to postulate (P.3), the inequality $\left|J_{v,\mathrm{ideal}}\right|>\left|J_{v,\mathrm{actual}}\right|$ must be valid at any time. However,$\;\left|J_{v,\mathrm{actual}}\right|$ cannot be lower than $\left|J_{v,\mathrm{ideal}}\right|$ when $\frac{\Delta P}{\Delta \pi }=1$ because $\left|J_{v,\mathrm{ideal}}\right|$ is already zero at that point. Since the value of $\left|J_{v,\mathrm{actual}}\right|$ must not be larger than that of $\left|J_{v,\mathrm{ideal}}\right|$, the best choice that can be made is to designate the value of $\left|J_{v,\mathrm{actual}}\right|$ as zero when $\frac{\Delta P}{\Delta \pi }=1$. However, the conclusion that $\left|J_{v,\mathrm{actual}}\right|=0$ at $\frac{\Delta P}{\Delta \pi }=1$ reveals another anomaly: namely, $\frac{\Delta P}{\Delta \pi }=1$. $\frac{S_{P}}{S_{\pi }}$ must become one in order to make $\left|J_{v,\mathrm{actual}}\right|=0$. That is, $S_{P}=S_{\pi }$ when $\frac{\Delta P}{\Delta \pi }=1$. This result also deviates from the criteria shown in Equation (25), which state that $S_{P}>S_{\pi }$ when $J_{v}=0$. This means that a dilemma inevitably occurs in the actual system when $\frac{\Delta P}{\Delta \pi }=1$. Hence, to avoid this dilemma, the current study does not define the value of $\left|J_{v,\mathrm{actual}}\right|$ when $\frac{\Delta P}{\Delta \pi }=1$. That is, the value of the water flux when $\frac{\Delta P}{\Delta \pi }=1$ remains unknown. Instead, this study assumes that $\left|J_{v,\mathrm{actual}}\right|\to 0$ because $\frac{\Delta P}{\Delta \pi }\to 0$ along with the change of $\left|J_{v,\mathrm{ideal}}\right|$, as a result of postulates (P.1) and (P.3).

**Figure 5:**
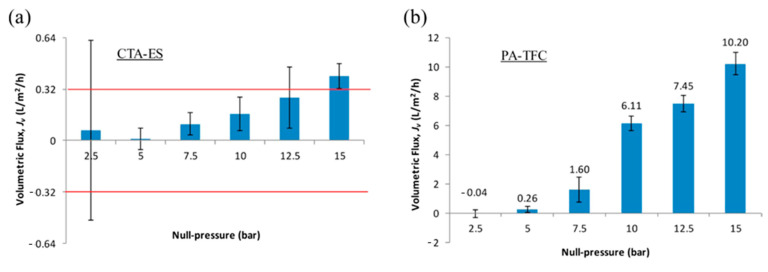
Volumetric fluxes under the null-pressure condition (i.e.,$\frac{\Delta P}{\Delta \pi }=1$) for (**a**) the cellulose triacetate (CTA) membrane and (**b**) the thin-film composite (TFC) membrane with relevant standard deviations. While the water flux of CTA under the null-pressure condition is within the admittable error range of zero water flux, that of the TFC deviates far from the error range [57]. Copyright 2018, American Chemical Society.

Concluding that $\left|J_{v,\mathrm{actual}}\right|$ approaches zero infinitesimally but never actually becomes zero is critical. Note that the fact that $J_{v}$ cannot be defined at $\frac{\Delta P}{\Delta \pi }=1$ does not imply that the value of $J_{v}$ at the point is not “measurable.” There are certainly some measured values for $J_{v}$ when $\frac{\Delta P}{\Delta \pi }=1$. However, the measured values of $J_{v}$ when $\frac{\Delta P}{\Delta \pi }=1$ significantly differ in accordance with the membrane types and do not exhibit a generalizable consistency. For example, one of the aforementioned studies has observed that the divide between the values of $J_{v}$ when $\frac{\Delta P}{\Delta \pi }=1$ could be more than tenfold, depending on the membrane types [57]. The membrane types given in the previous study are cellulose triacetate (CTA) and thin-film composite (TFC) membranes. In the study, the water flux values of the CTA membrane are slightly more or less than zero until the magnitude of both driving pressures reaches 15 bar with the constraint of $\frac{\Delta P}{\Delta \pi }=1$. However, the water flux values of the TFC membrane are at least five times higher than those of the CTA membrane in most of the magnitudes of driving pressures (see Figure 5). The study found that the divide between CTA and TFC membranes is attributed to different vulnerabilities of hydraulic pressure. In other words, the value of $J_{v}$ when $\frac{\Delta P}{\Delta \pi }=1$ does not depend on the amount of hydraulic pressure or osmotic pressure but on the physical robustness of the membranes when resisting hydraulic pressure. After all, the value of $J_{v}$ when $\frac{\Delta P}{\Delta \pi }=1$ is not theoretically generalizable for a combination of driving pressures so that the value of $J_{v}$ at $\frac{\Delta P}{\Delta \pi }=1$ is not definable. It is possible to measure the independent value of $J_{v}$ by considering the physical characteristics of membranes; however, it still cannot be ensured that the value of $J_{v}$ would go to zero. In Section 3.4, this topic is addressed with a more detailed explanation.

**Figure 6:**
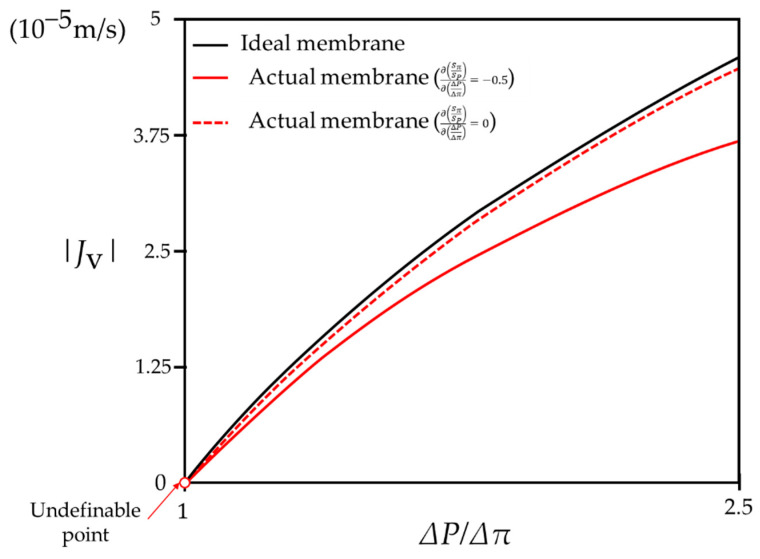
A plot with respect to the relationship between the ratio of the bulk driving pressures $\left(\frac{\Delta P}{\Delta \pi }\right)$ and the absolute value of the water flux $\left(J_{v}\right)$ in the RO mode. Since the sign of the RO water flux is defined as a minus in the current study, the absolute value bars are appended to $J_{v}$. The water flux is determined with the equation $J_{v}=-k\ln\left(\frac{\Delta P_{\mathrm{pse}}}{\Delta \pi_{\mathrm{pse}}}\right)$ by setting the value of $k=5*10^{-5}\;m/s$. The solid red curve indicates the change of $J_{v}$ when an actual membrane is employed with the condition of $\frac{\partial \left(\frac{S_{\pi }}{S_{P}}\right)}{\partial \left(\frac{\Delta P}{\Delta \pi }\right)}=-0.5$. The dashed red curve indicates the change of $J_{v}$ when an actual membrane is employed with the condition of $\frac{\partial \left(\frac{S_{\pi }}{S_{P}}\right)}{\partial \left(\frac{\Delta P}{\Delta \pi }\right)}=0$. In both cases, the value of $\frac{S_{\pi }}{S_{P}}=0.98$ at $\frac{\Delta P}{\Delta \pi }=1.05$. The black curve indicates the change of $J_{v}$ when an ideal membrane is employed. The value of the water flux is undefinable when $\frac{\Delta P}{\Delta \pi }=1$ due to the dilemma between postulate (P.3) and Equation (25).

Figure 6 illustrates the changes in the water flux according to the ratio of driving pressures in the RO mode with the condition of $k=5*10^{-5}m/s$. In the figure, $\frac{\Delta P}{\Delta \pi }$ ranges from 1 to 2.5, where the value of ΔP becomes 62.5 bar if Δπ is assumed to be 25 bar. The solid red curve indicates the change of $J_{v}$ when an actual membrane is employed with the condition of $\frac{\partial \left(\frac{S_{\pi }}{S_{P}}\right)}{\partial \left(\frac{\Delta P}{\Delta \pi }\right)}=-0.5$. The dashed red curve indicates the change of $J_{v}$ when an actual membrane is employed with the condition of $\frac{\partial \left(\frac{S_{\pi }}{S_{P}}\right)}{\partial \left(\frac{\Delta P}{\Delta \pi }\right)}=0$. In both cases, the value of $\frac{S_{\pi }}{S_{P}}=0.98$ at $\frac{\Delta P}{\Delta \pi }=1.05$. In Figure 6, the value of the water flux when $\frac{\Delta P}{\Delta \pi }=1$ is not defined, as described above, and the plot with respect to the water flux is monotonically increasing. On the other hand, in FO and PRO modes, the plot with respect to the water flux is monotonically decreasing, as shown in Figure 2. Such tendencies are based on the aforementioned postulate (P.1). To fix the plots with respect to the water flux as monotonic functions, the constraint regarding the similarity coefficient ratio, which can be defined as $\frac{S_{P}}{S_{\pi }}$, needs to be investigated.

### 3.4. Hypothesis for the Water Flux in the Transition Region between FO/PRO and RO

**Figure 7:**
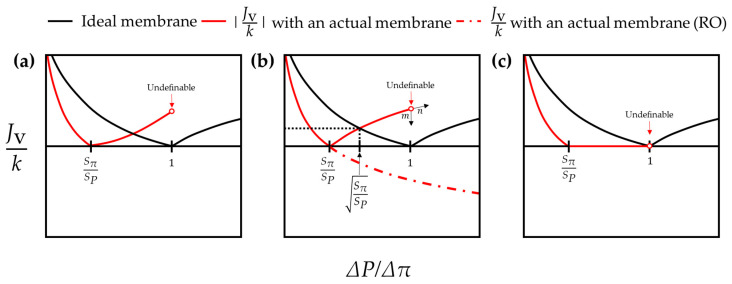
Plots representing the hypothetical tendencies of the water flux $\left(J_{v}\right)$ with respect to the driving pressures within the range of $\frac{S_{\pi }}{S_{P}}<\frac{\Delta P}{\Delta \pi }<1$. In (**a**), the value of $J_{v}$ rebounds and increases in FO/PRO modes. In contrast, $J_{v}$ enters the region of the RO mode in (**b**). Lastly, (**c**) illustrates the tendency that $J_{v}$ converges to zero.

Thus far, the overall tendencies of FO/PRO and RO modes have been investigated. However, a problem arises as soon as $\frac{\Delta P}{\Delta \pi }$ enters the range between $\frac{S_{\pi }}{S_{P}}$ and one. Given that $S_{P}>S_{\pi }$ in FO and PRO modes and $S_{P}<S_{\pi }$ in the RO mode, there might be a region in which the tendency in the similarity coefficient ratio is drastically converted. However, it is not easy to track the change in similarity coefficients because the actual membrane-based desalting process is neither FO/PRO nor RO in the region in which $\frac{\Delta P}{\Delta \pi }$ ranges from $\frac{S_{\pi }}{S_{P}}$ to one. Therefore, in the current study, the procedure for verifying the tendency in the given range should depend on indirect arguments based on the preceding postulates rather than mathematical proof. As shown in Figure 7, there are three other options for $J_{v,\mathrm{actual}}$ after entering the range of $\frac{S_{\pi }}{S_{P}}<\frac{\Delta P}{\Delta \pi }<1$. These three other options are:

(i.) Figure 7a. The direction of the water flux is not reversed and the absolute value of the water flux gradually increases as $\frac{\Delta P}{\Delta \pi }$ approaches one;
(ii.) Figure 7b. The water flux continually decreases so that the direction of the water transport gets reversed and the absolute value of the water flux gradually increases as $\frac{\Delta P}{\Delta \pi }$ approaches one; and
(iii.) Figure 7c. The water flux converges to zero and such a tendency is sustained.

First, (i) does not simultaneously comply with postulates (P.1) and (P.3). The violation of postulate (P.1) results from the increase in the water flux after a point at which $J_{v,\mathrm{actual}}=0$. In spite of postulate (P.1), once the water flux starts to increase, it must keep increasing or, at least, must remain the same because the water flux is a monotonic function. In that case, however, the water flux must exceed the value of the water flux made by the ideal membrane. At that point, the tendency of the water flux violates postulate (P.3). Therefore, (i) cannot be accepted.

On the other hand, (ii) does not indicate any problems within the range of $\frac{S_{\pi }}{S_{P}}<\frac{\Delta P}{\Delta \pi }<1$. Since the water transport direction in the actual system gets reversed after entering $\frac{S_{\pi }}{S_{P}}<\frac{\Delta P}{\Delta \pi }<1$, a comparison regarding the values of the water flux must be made in the actual RO mode and the ideal FO/PRO modes. Therefore, the tendency due to the absolute value of the water flux with the actual membrane after a point at which $\frac{\Delta P}{\Delta \pi }=\sqrt{\frac{S_{\pi }}{S_{P}}}$ is not problematic. However, a dilemma occurs as the water flux approaches $\frac{\Delta P}{\Delta \pi }=1$. Setting aside the fact that the water flux is undefinable when $\frac{\Delta P}{\Delta \pi }=1$, the water flux cannot be accurately determined even when $\frac{\Delta P}{\Delta \pi }>1$. For example, if the water flux tends to change in the direction of “m,” as marked in Figure 7b, then the overall tendency violates postulate (P.1) after all. In contrast, if the water flux tends to change in the direction of “n,” as it does within the range of $\frac{S_{\pi }}{S_{P}}<\frac{\Delta P}{\Delta \pi }<1$, then the given plot violates postulate (P.3). In either case, (ii) cannot be compatible with the preceding postulates.

Unlike (i) and (ii), (iii) does not breach any postulates. Once a given system fulfills the condition of $\frac{\partial \left(\frac{S_{P}}{S_{\pi }}\right)}{\partial \left(\frac{\Delta P}{\Delta \pi }\right)}=-\frac{\Delta \pi^{2}-\Delta \pi \Delta \pi_{\delta }}{\Delta P^{2}}$, (iii) is acceptable. Note that, in this case, $\frac{\partial \left(\frac{S_{P}}{S_{\pi }}\right)}{\partial \left(\frac{\Delta P}{\Delta \pi }\right)}$ becomes $-\frac{\Delta \pi^{2}}{\Delta P^{2}}$ because the condition of $J_{v,\mathrm{actual}}=0$ means $\Delta \pi_{\delta }=0$ (see Section 3.1). In the end, (iii) may be theoretically acceptable as the tendency of $J_{v,\mathrm{actual}}$ within the range of $\frac{S_{\pi }}{S_{P}}<\frac{\Delta P}{\Delta \pi }<1$. If this provisional theory is right, then the change in the similarity coefficient ratio within this range is reciprocal to the minus of the square of the ratio of the bulk driving pressures.

Although the case of (iii) may logically be acceptable in theory, questions still remain. For instance, one wonders why the value of the water flux can be maintained as zero despite changes to driving pressures. One also wonders how the one point at which $J_{v,\mathrm{actual}}=0$ can be chosen, as shown in Figure 2. To answer these questions, it is necessary to go back to Equation (3), which is the starting point of the current study. From the outset, this study has focused on phenomena that occur in the boundary layer of a membrane on the more concentrated side. According to Equation (7), the concentration polarization in the boundary layer serves to determine the overall direction of the water flux. All the concentration polarization phenomena result from changes within the boundary layer, which is located outside of a membrane. In other words, transport phenomena inside a membrane cannot be detected using equations derived from Equation (3). If $\frac{\Delta P}{\Delta \pi }$ is significantly small or large (i.e., $\frac{\Delta P}{\Delta \pi }<\frac{S_{\pi }}{S_{P}}$ and $\frac{\Delta P}{\Delta \pi }>1$), then either Δπ or ΔP is predominant for determining the tendencies of water transport in comparison to membrane parameters. Therefore, the preceding equations based on Equation (3) are reasonable. However, when $\frac{\Delta P}{\Delta \pi }$ enters the range of $\frac{S_{\pi }}{S_{P}}<\frac{\Delta P}{\Delta \pi }<1$, the equations based on membrane parameters become dominant.

Let us utilize the salt flux, $J_{c}$, to elucidate the difference within the range of $\frac{S_{\pi }}{S_{P}}<\frac{\Delta P}{\Delta \pi }<1$ between the equations based on Equation (3) and the equations based on membrane parameters. It is widely known that the salt flux is deeply related to the tendency of the water flux owing to the presence of hydration phenomena [59]. As mentioned earlier, the salt flux in the boundary layer is expressed as $J_{c}=J_{v}C_{l}$ [54]. That is, $J_{c}$ naturally becomes zero when $J_{v}=0$. In practice, however, the value of $J_{c}$ does not become zero even when $J_{v}=0$. A previous study [54] formulated the total salt flux in FO and PRO modes as follows:

(44) $$-J_{c}=\beta_{\mathrm{ov}}\left\{C_{h,b}-C_{l}\exp\left(J_{v}\left[\frac{1}{k_{l}}+\frac{1}{k_{h}}+\frac{S}{D_{C}}\right]\right)\right\}.$$

Here, $\beta_{\mathrm{ov}}$ is the diffusive plus convective mass transfer coefficient applied to the salt flux; $k_{l}$ is the diffusive mass transfer coefficient in the less concentrated side (the feed side of FO and PRO modes); $k_{h}$ is the diffusive mass transfer coefficient in the more concentrated side (the draw side of FO and PRO modes); and S is the structure parameter of a membrane. Note that the minus sign of $J_{c}$ in Equation (44) reflects the opposing direction of the salt flux with respect to the water flux in FO and PRO modes. According to the corresponding study, $\beta_{ov}$ is expanded as follows:

(45) $$\frac{1}{\beta_{\mathrm{ov}}}=\frac{1-\exp\left(\frac{J_{v}}{k_{h}}\right)}{J_{v}}+\left(\frac{1-\exp\left(\frac{J_{v}S}{D_{C}}\right)}{J_{v}}+\frac{\exp\left(\frac{J_{v}S}{D_{C}}\right)\left(1-\exp\left(\frac{J_{v}}{k_{l}}\right)\right)}{J_{v}}-\frac{1}{B}\right)\exp\left(\frac{J_{v}}{k_{h}}\right).$$

Here, B is the salt permeability of a membrane. If the value of each term in $\beta_{\mathrm{ov}}$ is approximated when $J_{v}\to 0$, then $\frac{1-\exp\left(\frac{J_{v}}{t}\right)}{J_{v}}$ goes to $-\frac{1}{t}$ and $\exp\left(\frac{J_{v}}{t}\right)$ goes to 1. Here, t represents one of the membrane parameters shown in Equation (45). Then, when $J_{v}\to 0$, Equation (45) becomes:

(46) $$\frac{1}{\beta_{\mathrm{ov}}}\approx -\frac{1}{k_{h}}-\frac{S}{D_{C}}-\frac{1}{k_{l}}-\frac{1}{B}.$$

In addition, $\exp\left(J_{v}\left[\frac{1}{k_{l}}+\frac{1}{k_{h}}+\frac{S}{D_{C}}\right]\right)\to 1$ when $J_{v}\to 0$ in Equation (44). Thus, by incorporating it with Equation (46), Equation (44) can be approximated as follows when $J_{v}\to 0$:

(47) $$J_{c}\approx \frac{\left(C_{h,b}-C_{l}\right)}{\frac{1}{k_{h}}+\frac{S}{D_{C}}+\frac{1}{k_{l}}+\frac{1}{B}}.$$

As shown in Equation (47), the value of $J_{c}$ never becomes zero, unless $C_{h,b}=C_{l}$. As salt molecules are transported across a membrane even when $J_{v}\to 0$, the water flux might be affected, to some extent, in return because of the hydration phenomena described above. Consequently, the presence of a membrane leads to variations in the water flux even though the bulk driving pressures theoretically allow the water flux of a system to be zero. In this context, the $\frac{S_{\pi }}{S_{P}}<\frac{\Delta P}{\Delta \pi }<1$ range, which is located between the FO/PRO and RO modes, can be called the “transition” region.

Although the equations newly established in the current study do not provide researchers with certain information with respect to the $\frac{S_{\pi }}{S_{P}}<\frac{\Delta P}{\Delta \pi }<1$ range, these new concepts may shed light on parts that cannot be theoretically interpreted by conventional models. There are definite advantages and drawbacks when the solute concentration is set as the transported variable. The biggest advantage is, by far, the fact that membrane parameters can be used freely. Most of the extant membrane parameters are based on a system in which the transported variable is determined as the solute concentration and the number of membrane parameters related to energy is very limited. In the same manner, issues caused by energy cannot be diagnosed or treated using conventional models. For example, the degree of membrane vulnerability to external hydraulic pressure, which was mentioned in the previous subsection, is very challenging to quantify theoretically using conventional models. Furthermore, it is difficult to find an exact relation between Δπ and ΔP without taking energy loss of ΔP into consideration. As mentioned above, the main contributing factors of ΔP energy loss are operational parameters such as crossflow velocity or channel height. Therefore, to find an accurate relation between Δπ and ΔP, the scope of the investigation should be broadened, even to a whole process.

Now, recall the two abovementioned questions. The first question can be answered quite simply. In order to find the exact values of the water flux within the $\frac{S_{\pi }}{S_{P}}<\frac{\Delta P}{\Delta \pi }<1$ range, it is necessary to investigate membrane parameters rather than components of the boundary layer—namely, the fact that the water flux values within the $\frac{S_{\pi }}{S_{P}}<\frac{\Delta P}{\Delta \pi }<1$ range are zero must be inferred from information regarding the boundary layer system. The practical values of the water flux can sufficiently be changed if membrane parameters are taken into consideration. Needless to say, the degree of change in the water flux depends on the real values of membrane parameters. That is, the water flux within the $\frac{S_{\pi }}{S_{P}}<\frac{\Delta P}{\Delta \pi }<1$ range can vary as a result of changes to membrane parameters. Such fluctuation is not a generalizable phenomenon and, thus, in answer to the second question, the point at which $J_{v,\mathrm{actual}}=0$ within $\frac{S_{\pi }}{S_{P}}<\frac{\Delta P}{\Delta \pi }<1$ cannot be chosen theoretically.

According to the equations formulated to describe the boundary layer, $J_{v,\mathrm{actual}}$ is sustained as zero in the membrane-dominant region. When the membrane parameters are taken into account, however, $J_{v,\mathrm{actual}}$ can fluctuate to some extent. A specific point marked with dashed lines in an RO region indicates a point at which the water flux of RO can maximally be achieved using current technology and usual seawater. According to a previous study [60], the maximum hydraulic pressure that can be applied to conventional RO processes is around 80 bar. Likewise, the practical water flux limit is also designated in this figure, as discussed earlier.

**Figure 8:**
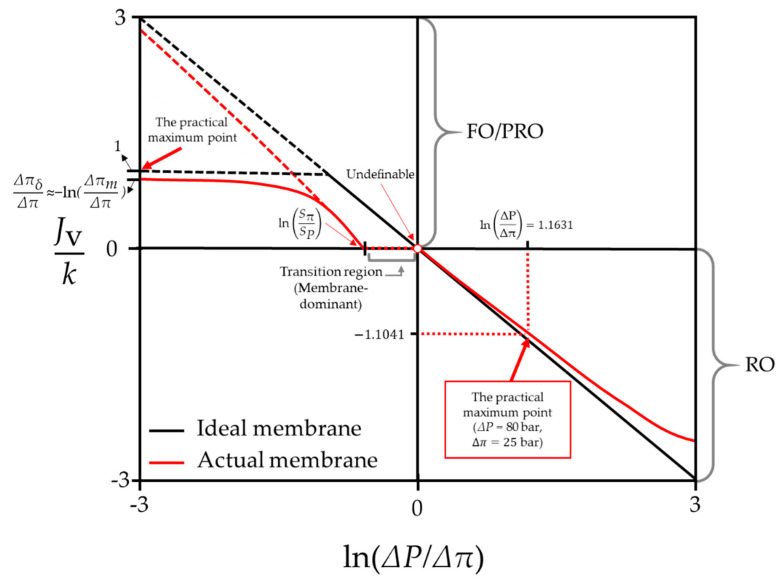
Summing up the overall tendency of the water flux in membrane-based desalting systems in accordance with the conditions given in Table 2. In this figure, for convenience, the value of the water flux is nondimensionalized with the mass transfer coefficient, k. The straight black line indicates changes in the dimensionless water flux made by the ideal membrane $\left(\frac{J_{v,\mathrm{ideal}}}{k}\right)$, the red curve indicates changes in the dimensionless water flux made by an actual membrane $\left(\frac{J_{v,\mathrm{actual}}}{k}\right)$, and the dashed red line, within $\ln\left(\frac{S_{\pi }}{S_{P}}\right)<\ln\left(\frac{\Delta P}{\Delta \pi }\right)<0$, indicates a “transition” (membrane-dominant) region.

Finally, the relation between the bulk osmotic pressure and the bulk hydraulic pressure can be entirely traced to changes in the water flux. Figure 8 represents the overall water flux tendency in all types of membrane-based desalting systems. To observe the overall tendencies of $J_{v}$, the range of $\frac{\Delta P}{\Delta \pi }$ is set as (−3, 3). Conditions required to plot Figure 8 are tabulated in Table 2. In Figure 8, the transition region between the FO/PRO and RO modes is marked with a dashed line. A point marked with dashed lines in an RO region of Figure 8 represents the maximum water flux value in seawater RO that can be produced in practice. According to a previous study [60], the maximum hydraulic pressure that can be applied to conventional RO processes is around 80 bar and the osmotic pressure of $C_{h}$, which was assumed to be 35,000 ppm in the current study, is estimated around 25 bar. Therefore, the value of $\ln\left(\frac{\Delta P}{\Delta \pi }\right)$ at that point can be considered to be slightly larger than one.

As expected, $J_{v,\mathrm{actual}}$ in FO and PRO modes is significantly lower than $J_{v,\mathrm{ideal}}$. On the other hand, $J_{v,\mathrm{actual}}$ in the RO mode does not show a huge difference in comparison to $J_{v,\mathrm{ideal}}$ when $\ln\left(\frac{\Delta P}{\Delta \pi }\right)$ is not high. The opposite trend between FO/PRO and RO modes reveals the advantages of an RO process over FO and PRO processes.

### 3.5. Practical Implications of Theoretical Analyses with Respect to Driving Pressures

Table 3 represents all constraints for implementing $J_{v,\mathrm{actual}}$ in each membrane-based desalting process. Except for the transition region between FO/PRO and RO processes, $\frac{S_{\pi }}{S_{P}}<\frac{\Delta P}{\Delta \pi }<1$, the overall tendencies of membrane-based desalting processes can be illustrated, together with the constraints in Table 3. In other words, the given systems can be operated freely if the constraints below are fulfilled.

Although the forms of the constraints for FO/PRO and RO modes look similar, the actual situations for each process vary greatly. In the RO mode, when $\frac{\Delta P}{\Delta \pi }$ is close to one, the values of $\frac{S_{P}}{S_{\pi }}$ must fall into a narrow range for fulfilling the first and second constraints simultaneously. That is, the value of $\frac{S_{P}}{S_{\pi }}$ must be lower than one owing to the second constraint, but it must be sufficiently large due to the first constraint. Therefore, the value of $J_{v,\mathrm{actual}}$ in the RO mode is maintained close to the value of $J_{v,\mathrm{ideal}}$ when $\frac{\Delta P}{\Delta \pi }$ is not big enough. On the other hand, regardless of the increase or decrease in $\frac{S_{P}}{S_{\pi }}$, the discrepancy between $J_{v,\mathrm{ideal}}$ and $J_{v,\mathrm{actual}}$ is significantly large in FO/PRO modes. To operate the processes, however, the values of $\frac{S_{P}}{S_{\pi }}$ can be designated relatively freely.

In short, the advantage of FO/PRO modes is that the operation of these processes is straightforward. However, there is a drawback to these processes in that $J_{v,\mathrm{actual}}$ is inherently far lower than $J_{v,\mathrm{ideal}}$. On the other hand, operating the RO process requires delicate settings for determining the values of similarity coefficients. Once the RO process begins operation, however, performance is very high in comparison to the FO/PRO processes. Practically speaking, such a difference is one of the reasons why the RO process is more advantageous for commercialization. Although setting the process conditions is relatively tough, the performance of the RO process is clearly better than that of FO/PRO processes.

**Figure 9:**
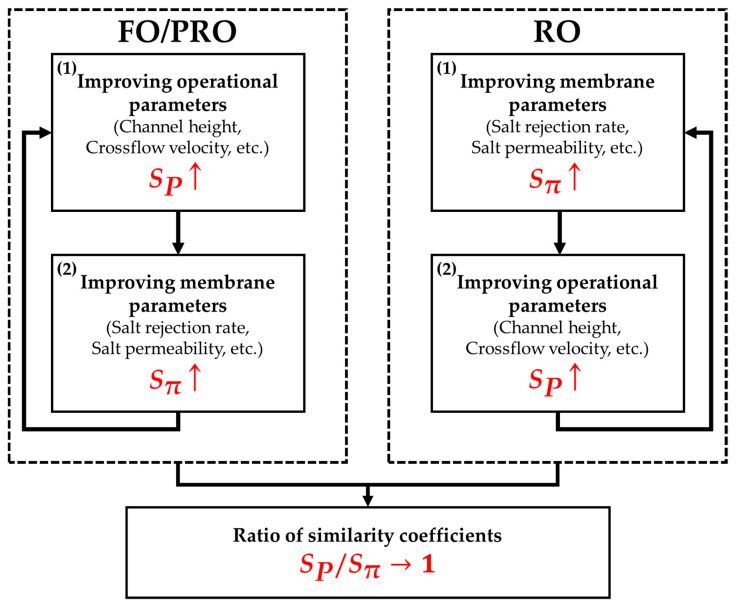
A chart visually representing the ideal sequences for improving the performance of each membrane-based desalting process. To produce as much water as possible (i.e., making $\frac{S_{P}}{S_{\pi }}$ close to one) with the smallest energy loss (i.e., making both $S_{P}$ and $S_{\pi }$ close to one), each process must take different steps due to the second constraint in Table 3. In FO/PRO modes, (1) the operational parameters that mostly determine the value of $S_{P}$ should be improved before (2) the membrane parameters of. However, in the RO mode, (1) the membrane parameters that mostly determine the value of $S_{\pi }$ should be improved before (2) the operational parameters.

Another important practical implication of the current study is that improving only one of the membrane or operational parameters is not advisable. As mentioned earlier, the common final goal of membrane-based desalting processes is to produce water volume that is as large as possible with the smallest energy loss. Therefore, for the best performance of membrane-based desalting processes, $S_{P}$, $S_{\pi }$, and $\frac{S_{P}}{S_{\pi }}$ should be around the value of one at the same time. However, since the inequalities between similarity coefficients are different in accordance with the types of membrane-based desalting processes, as given in Table 3, optimization work for parameters should be conducted in a distinct order. For FO/PRO modes, it is recommended to first improve the value of $S_{P}$ by optimizing operational parameters because $S_{\pi }$ has the innate limit in that its value cannot exceed $S_{P}$. Subsequently, $S_{\pi }$ should catch up to $S_{P}$. If not, $\frac{S_{P}}{S_{\pi }}$ naturally increases so that the value of $J_{v}$ decreases. This is the reason why the two similarity coefficients need to be regarded as single coefficients that are coupled to one another rather than as entirely separate coefficients. If only the improvement of each singular coefficient is sought, then the overall performance of a process could be degraded. Likewise, a suitable optimization sequence for the RO mode can be determined. For the RO mode, the improvement of $S_{\pi }$ should precede the improvement of $S_{P}$, and $S_{P}$ should later catch up to $S_{\pi }$. This cycle of similarity coefficients can be continued until the value of $\frac{S_{P}}{S_{\pi }}$ becomes slightly larger or less than one. All aforementioned procedures are visually summarized in Figure 9. In accordance with the type of membrane-based desalting processes, an appropriate optimization sequence should be selected.

## 4. Conclusions

There has long been a need for a formula that captures the clear relation between the driving pressures that operate in membrane-based desalting processes such as FO, PRO, and RO. In this respect, with the energy loss of each driving pressure, this study reveals the actual mathematical relation between the driving pressures of membrane-based desalting processes, the bulk osmotic pressure difference (Δπ), and the bulk hydraulic pressure (ΔP). To find the relation, this study first suggested self-evident postulates based on the energy conservation rule and the relation between the ideal membrane and actual membranes. This study then redefined the conventional water transport model by transforming the transported variable of a system from solute concentration to driving pressures and by embodying the relationship between driving pressures using new variables and coefficients. Herein, these new variables stand for intangible pseudo-driving pressures $(\Delta \pi_{\mathrm{pse}}$ and $\Delta P_{\mathrm{pse}})$, while the new coefficients stand for similarity coefficients $(S_{\pi }$ and $S_{P})$ that bridge the pseudo-driving pressures and the bulk driving pressures. According to the definition of similarity coefficients, $\Delta P_{\mathrm{pse}}=S_{P}\Delta P$ and $\Delta \pi_{\mathrm{pse}}=S_{\pi }\Delta \pi$. When $\Delta \pi_{\mathrm{pse}}>\Delta P_{\mathrm{pse}}$, a given membrane-based desalting system becomes either an FO or a PRO process. Meanwhile, the case in which $\Delta \pi_{\mathrm{pse}}<\Delta P_{\mathrm{pse}}$ represents the RO process. If $\Delta \pi_{\mathrm{pse}}=\Delta P_{\mathrm{pse}}$, then there is no water flux ($J_{v}$) in the system. In association with the fundamental postulates and the relation established for driving pressures, then tendencies of the water flux according to the change of $\frac{\Delta P}{\Delta \pi }$ were analyzed in this study. These analyses are summarized here as follows:

(I) Δπ and ΔP are related via the osmotic pressure difference in the boundary layer of the more concentrated side of a system, $\Delta \pi_{\delta }$. When a given process is operated in FO/PRO modes, then $\Delta \pi =\frac{S_{P}}{S_{\pi }}\Delta P+\Delta \pi_{\delta }$. On the other hand, $\Delta \pi =\frac{S_{P}}{S_{\pi }}\Delta P-\Delta \pi_{\delta }$ if the given process is RO.
(II) Since $\Delta \pi_{\mathrm{pse}}>\Delta P_{\mathrm{pse}}$ in FO/PRO modes and $\Delta \pi_{\mathrm{pse}}<\Delta P_{\mathrm{pse}}$ in the RO mode, this means that $1>\frac{S_{P}\Delta P}{S_{\pi }\Delta \pi }$ for FO/PRO modes and $1<\frac{S_{P}\Delta P}{S_{\pi }\Delta \pi }$ for the RO mode. In addition, based on the postulate that specifies that the performance of actual membranes never exceeds that of the ideal membrane, $S_{\pi }<S_{P}$ in FO/PRO modes and $S_{\pi }>S_{P}$ in the RO mode. This contrast between FO/PRO and RO modes is critical for optimizing process parameters.
(III) The point at which $J_{v}=0$ always belongs to the FO/PRO region due to the reflection coefficient that states that $S_{\pi }<S_{P}$ when $J_{v}=0$. In other words, $J_{v}$ never becomes zero in the RO mode, theoretically.
(IV) There can exist a practical water flux limit for FO and PRO processes, unless severe dilutive external concentration polarization is assumed.
(V) When $\frac{\Delta P}{\Delta \pi }=1$, the value of the water flux made by the actual membranes cannot be defined because the value of the water flux at that point does not comply with the fundamental postulates.
(VI) Given that $J_{v}$ always monotonically increases or decreases according to $\frac{\Delta P}{\Delta \pi }$, the value of $\frac{\partial \left(\frac{S_{P}}{S_{\pi }}\right)}{\partial \left(\frac{\Delta P}{\Delta \pi }\right)}$ in desalting systems must be equal to or larger than a specific negative value (see Table 3).
(VII) Within the range of $\frac{S_{\pi }}{S_{P}}<\frac{\Delta P}{\Delta \pi }<1$, in principle, the value of $J_{v}$ is maintained as zero according to the preceding postulates. However, the practical values of $J_{v}$ within the range fluctuate to some extent because of the presence of membrane parameters.

Based on the analyses presented in this study, the advantages and the drawbacks of the FO/PRO and RO processes can also be discussed. The actual membrane-based desalting processes should follow the constraints shown in Table 3. Fulfilling the constraints for the RO process is relatively difficult in comparison with FO/PRO processes. However, once the operation of the process begins, the RO process outperforms FO/PRO processes. That is, even in theory, it is more advantageous to commercialize the RO process rather than FO/PRO processes if a well-controlled system is implemented.

From a practical perspective, the most important lesson of the current study is that augmenting only $S_{\pi }$ or $S_{P}$ for the performance of membrane-based desalting systems is not that helpful. In other words, optimizing only one out of all membrane and operational parameters does not have a substantial impact on desalting systems. Occasionally, improving only one of the parameters might actually result in worse process performance. To significantly enhance the performance of membrane-based desalting systems, both membrane and operational parameters should be improved using suitable optimization sequences. Consequently, it could be said that the overall performance of membrane-based desalting systems hinges on the difference between $S_{\pi }$ and $S_{P}$.

## Figures and Tables

**Table 1:** This table consists of the ratios of the maximal water flux ($J_{v,\max}$ ) from each paper to the mass transfer coefficient (k ) within the boundary layer of the more concentrated side.

Value of $\frac{J_{v,max}}{k}$ (FO)	Value of $\frac{J_{v,max}}{k}$ (PRO)	Reference
0 (Assumed that k→∞)		[52]
0.0823	0.0724	[53]
0.3874	0.6854	[50]
0.34	-	[54]
0.2830	0.8852	[55]
-	0.4329	[56]

**Table 2:** The conditions utilized to plot Figure 8.

Process Types	Conditions	Condition Setting
FO/PRO	${\left(\frac{S_{P}}{S_{\pi }}\right)}_{\frac{\Delta P}{\Delta \pi }=0.05}$	1.2
	$\frac{\partial \left(\frac{S_{P}}{S_{\pi }}\right)}{\partial \left(\frac{\Delta P}{\Delta \pi }\right)}$	1.0
	${\left(\frac{\Delta P}{\Delta \pi }\right)}_{J_{v}=0}$	0.579
	${\left(J_{v}\right)}_{\frac{S_{\pi }}{S_{P}}<\frac{\Delta P}{\Delta \pi }<1}$	0
RO	${\left(\frac{S_{P}}{S_{\pi }}\right)}_{\frac{\Delta P}{\Delta \pi }=1.05}$	0.99
	$\frac{\partial \left(\frac{S_{P}}{S_{\pi }}\right)}{\partial \left(\frac{\Delta P}{\Delta \pi }\right)}$	−0.022

**Table 3:** The constraints required for actual membrane-based desalting systems.

Constraints	FO/PRO	RO
First constraint (Pseudo-Pressure)	$1>\frac{S_{P}}{S_{\pi }}\frac{\Delta P}{\Delta \pi }$ $\left(\Delta \pi_{\mathrm{pse}}>\Delta P_{\mathrm{pse}}\right)$	$1<\frac{S_{P}}{S_{\pi }}\frac{\Delta P}{\Delta \pi }$ $\left(\Delta \pi_{\mathrm{pse}}<\Delta P_{\mathrm{pse}}\right)$
Second constraint (Similarity)	$S_{\pi }<S_{P}$	$S_{\pi }>S_{P}$
Third constraint (Monotonic)	$\frac{\partial \left(\frac{S_{P}}{S_{\pi }}\right)}{\partial \left(\frac{\Delta P}{\Delta \pi }\right)}\geq -\frac{\Delta \pi^{2}-\Delta \pi \Delta \pi_{\delta }}{\Delta P^{2}}$	$\frac{\partial \left(\frac{S_{P}}{S_{\pi }}\right)}{\partial \left(\frac{\Delta P}{\Delta \pi }\right)}\geq -\frac{\Delta \pi^{2}+\Delta \pi \Delta \pi_{\delta }}{\Delta P^{2}}$

## Nomenclatures

B	The salt permeability of a semi-permeable membrane (g/mol s)
S	The structure parameter of a semi-permeable membrane (m)
R	Rejection rate of a semi-permeable membrane (-)
T	Temperature (K)
ΔP	External hydraulic pressure (Pa)
D	The diffusivities defined in membrane-based desalting systems (m^2^/s)
$T_{p}$	The diffusivities in membrane-based desalting systems when the transported variable is the driving pressures (m^2^/s)
k	The mass transfer coefficients in membrane-based desalting systems (m^3^/m^2^ s)
$S_{\pi }$	The similarity coefficients bridging the pseudo-osmotic pressures and the bulk osmotic pressures (-)
$S_{P}$	The similarity coefficients bridging the pseudo-osmotic pressures and the bulk osmotic pressures (-)
a	Arbitrary coefficients bridging the pseudo-driving pressures and the solute concentration (N m/mol)
X	Transported variables of membrane-based desalting systems
C	Solute concentration in membrane-based desalting systems (mol/m^3^)
DP	Driving pressures in membrane-based desalting systems (Pa)
y	Distance from the surface of a semi-permeable membrane (m)
Y	Dimensionless distance from the surface of a semi-permeable membrane to the end of a boundary layer (-)
$J_{v}$	Water flux in membrane-based desalting systems (m^3^/m^2^ s)
$J_{c}$	Salt flux in membrane-based desalting systems (g/m^2^ s)
**Greek symbols**	
π	Osmotic pressure (Pa)
δ	Length of a boundary layer in the more concentrated side of the membrane-based desalting systems (m)
α	Arbitrary pressure existing in the less concentrated side of membrane-based desalting systems (Pa)
σ	The reflection coefficient of membrane-based desalting systems (-)$\beta_{ov}$ The diffusive and convective mass transfer coefficient applied to the salt flux (g/mol s)
**Subscripts and superscripts**	
C	The transported variable of a system is the solute concentration
DP	The transported variable of a system is the driving pressure (specific energy)
h	A more concentrated side of membrane-based desalting systems
l	A less concentrated side of membrane-based desalting systems
m	The solute concentration at the semi-permeable membrane surface
b	The solute concentration in the bulk more concentrated region
pse	Pseudo-driving pressures
ideal	The ideal system with the ideal semi-permeable membrane
actual	The actual system with the actual semi-permeable membrane

## Appendix A. Justification for the Equality between the Concentration-Based Differential Equation and the Pressure-Based Differential Equation

As mentioned above, the differential equations that relate to solute concentration and driving pressures are interchangeable. The key to such interchangeability is the fact that the pressures of fluids can always be formulated by volume (V), number of molecules (n), and temperature (T). Thus, driving pressures of a membrane-based desalting system can be defined as follows:

(A1) DP=f(n,V,T).

Assuming that the temperature of the fluid is constant across an entire system, the function of DP changes into f(n,V). Here, DP is the intensive property and n and V are the extensive properties. The only way to express an intensive property using extensive properties is to mutually divide those extensive properties. Hence, DP can alternatively be defined as follows:

(A2) $$DP=f\left(n,V\right)=g\left(\frac{n}{V}\right)=g\left(C\right).$$

Equation (A2) indicates that DP is a function of the solute concentration. The most well-known functions for f and g are the equations of states, such as the ideal gas law and the van’t Hoff equation, respectively. For example, let DP be CRT according to the van’t Hoff equation; then Equation (3) becomes:

(A3) $$D_{i}^{*}\frac{d\left(DP/RT\right)}{dy}=\frac{D_{i}^{*}}{RT}\frac{d\left(DP\right)}{dy}=\frac{J_{v}}{RT}\left(DP-\alpha \right).$$

Since RT on both sides can be canceled, the form of the differential equation with respect to driving pressures becomes the same as that of the differential equation with respect to the solute concentration. Now that Equation (A3) is found to be the simplest differential equation with respect to driving pressures, this study only utilizes equation (A3). Needless to say, f(n,V,T) can alternatively become other types of equations that can relate driving pressures. For example, the Harmon Northrop Morse equation, an equation estimating osmotic pressure along with molality instead of molarity, can be applied if the solvent density is considered. Using the Harmon Northrop Morse equation, the osmotic pressure can be better approximated because molality is invariable along with the temperature. However, this study solely utilizes the van’t Hoff equation by assuming that the solvent density (i.e., the density of water) is constantly one. This is because changes in the solvent density should be taken into consideration when the Harmon Northrop Morse equation is used for relating the osmotic pressure to the hydraulic pressure. The use of the solvent density can lead to extremely complicated variations, which make the final result highly difficult to interpret. Therefore, the current study only uses the van’t Hoff equation by assuming that the density of the solvent is one.

Likewise, this study assumes that the activity coefficients of the solutions in the membrane-based desalting systems are always one. As mentioned above, the variations of the activity coefficient can make the overall contents difficult to interpret and increase the uncertainty of the results. In this regard, to clearly exhibit the implication of this study, the value of the activity coefficient is fixed as one.

## Appendix B. Brief Derivation for the Water Flux with Respect to Driving Pressures

Since the equality between the concentration-based differential equation and the pressure-based differential equation is shown in Appendix A, a brief derivation for the water flux with respect to driving pressures is all that is needed for now. The following equation is obtained when Equations (3), (4b), and $k_{DP}$ in Equation (5) are incorporated:

(A4) $$T_{p}\frac{d\left(DP\right)}{dy}=J_{v}\left(DP-\alpha \right).$$

Recall that $Y=\frac{y}{\delta }$. Then, Equation (A4) becomes:

(A5) $$\frac{T_{p}}{\delta }\frac{d\left(DP\right)}{dY}=k_{DP}\frac{d\left(DP\right)}{dY}=J_{v}\left(DP-\alpha \right).$$

Therefore, the following relation is valid according to Equation (A5):

(A6) $$k_{DP}\frac{d\left(DP\right)}{DP-\alpha }=J_{v}dY.$$

According to the boundary conditions set in Equation (6b), $DP=DP_{1}$ at Y=0 and $DP=DP_{2}$ at Y=1. By integrating both sides in Equation (A6) with the boundary conditions, Equation (8) is finally obtained as follows:

(A7) $$J_{v}=k_{DP}\ln\left(\frac{DP_{2}-\alpha }{DP_{1}-\alpha }\right)=-k_{DP}\ln\left(\frac{DP_{1}-\alpha }{DP_{2}-\alpha }\right).$$

## Appendix C. Brief Explanation on a Notation for the Mass Transfer Coefficient, k

To incorporate Equation (8) with Equations (10) and (11), Equation (8) must be manipulated as follows in order to add the power term $\frac{k_{DP}}{k_{C}}$:

(A8) $$J_{v,DP}=-k_{C}\left(\frac{k_{DP}}{k_{C}}\right)\ln\left(\frac{DP_{1}-\alpha }{DP_{2}-\alpha }\right).$$

Thus, Equation (A8) becomes:

(A9) $$J_{v,DP}=-k_{C}\ln\left(\frac{\Delta P_{\mathrm{pse}}}{\Delta \pi_{\mathrm{pse}}}\right).$$

As shown in Equation (A9), the mass transfer coefficient for $J_{v,DP}$ is also $k_{C}$, which was initially defined for a case in which a transported variable is the solute concentration. In the end, $k_{DP}$ becomes redundant when observing $J_{v}$, which is the reason why $k_{C}$ and $k_{DP}$ are unified as k.
